# Supplementary material for: Subcellular localization of biomolecules and drug distribution by high-definition ion beam imaging
Source: Nat Commun. 2021 Jul 30;12:4628. doi: 10.1038/s41467-021-24822-1 (PMC8324837; doi:10.1038/s41467-021-24822-1)
Supplement: Supplementary file 1 — Supplementary Information [file 41467_2021_24822_MOESM1_ESM.pdf]

Supplementary Materials for

Subcellular localization of biomolecules and drug distribution by high-definition ion beam imaging

Xavier Rovira-Clave[1]†, Sizun Jiang[1]†, Yunhao Bai[1], Bokai Zhu[1], Graham Barlow[1], Salil Bhate[1,2], Ahmet F. Coskun[1], Guojun Han[1], Chin-Min Kimmy Ho[1]¶, Chuck Hitzman[3], Shih-Yu Chen[1#]‡, Felice-Alessio Bava[1#]§ and Garry P. Nolan[1#]*.

† These authors contributed equally

[#] Co-senior authors

* Correspondence should be addressed to G.P.N. (gnolan@stanford.edu)

This PDF file includes:

Supplementary Figures 1 to 35

Supplementary Tables 1 to 4

Supplementary Notes 1 to 3

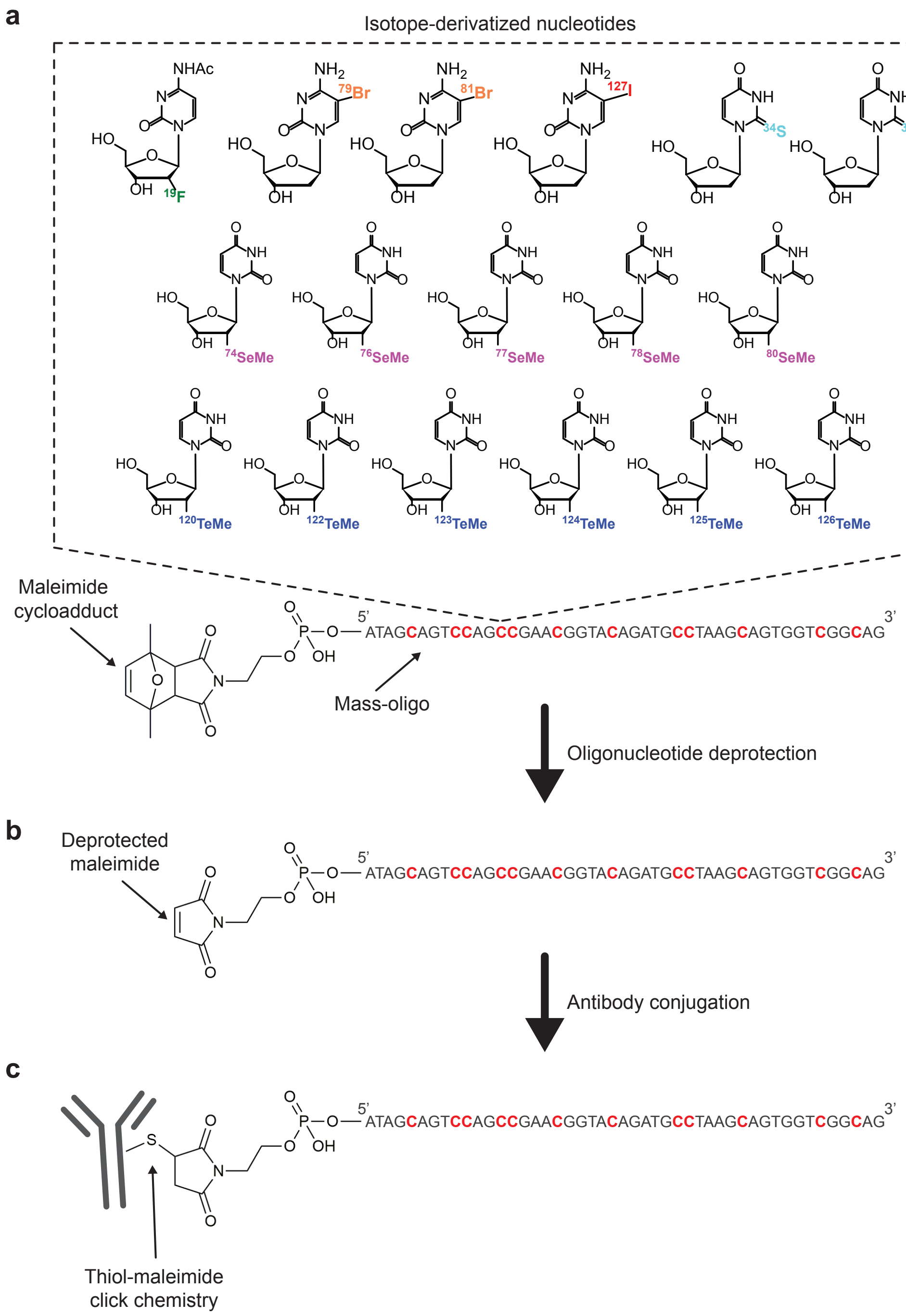

a
Isotope-derivatized nucleotides
Maleimide cycloadduct
Mass-oligo
5' ATAGCAGTCCAGCCGAACGGTACAGATGCCTAAGCAGTGGTCGGCAG 3'
Oligonucleotide deprotection
b
Deprotected maleimide
Antibody conjugation
c
Thiol-maleimide click chemistry

Supplementary Figure 1. Strategy for the synthesis and conjugation of mass-oligonucleotides to antibodies.

(**A**) Mass-oligonucleotides (Mass-oligo) were synthesized with internal isotope-derivatized nucleotides and a maleimide cycloadduct at the 5' position. Dashed box shows chemical structures of isotope-derivatized cytidines and thymidines that are commercially available or that can be synthesized with previously reported protocols (*1, 2*). In this study, we incorporated 12 fluorine, bromine, or iodine-derivatized cytidines (shown in red) into a 47-nucleotide long oligonucleotide. The DNA sequence was selected to minimize potential for binding to the human genome.

(**B**) Oligonucleotides were deprotected in toluene for 4 hours at 90 °C, washed in ethanol, lyophilized, and stored at -20 °C.

(**C**) The deprotected oligonucleotides were conjugated to partially reduced antibodies through thiol-maleimide click chemistry. Free oligonucleotides were removed via size-exclusion by centrifugation using a 50-KDa molecular weight cut-off filter.

Sample	Fixation and Permeabilization	Blocking	1ry antibody	2ry antibody
1	Methanol / Acetic acid (1:1; 10 min; -20°C)	PBS (1X)<br>Tween-20 (0.05%)<br>FBS (5%)	anti-dsDNA (1:500)	anti-mouse-Ax657 (1:2000)
2	Methanol / Acetic acid (1:1; 10 min; -20°C)	PBS (1X)<br>Tween-20 (0.05%)<br>FBS (5%)	anti-dsDNA-^{162}Dy (1:50)	anti-mouse-Ax657 (1:2000)
3	Methanol / Acetic acid (1:1; 10 min; -20°C)	PBS (1X)<br>BSA (0.5%)<br>EDTA (5 mM)<br>NaCl (0.5 M)<br>Salmon sperm DNA (200 μg/mL)<br>Human TruStain FcX (20 μg/mL)	anti-dsDNA-^{162}Dy (1:50)	anti-mouse-Ax657 (1:2000)
4	Methanol / Acetic acid (1:1; 10 min; -20°C)	PBS (1X)<br>Tween-20 (0.05%)<br>FBS (5%)	anti-dsDNA-^{127}I (1:50)	anti-mouse-Ax657 (1:2000)
5	Methanol / Acetic acid (1:1; 10 min; -20°C)	PBS (1X)<br>BSA (0.5%)<br>EDTA (5 mM)<br>NaCl (0.5 M)<br>Salmon sperm DNA (200 μg/mL)<br>Human TruStain FcX (20 μg/mL)	anti-dsDNA-^{127}I (1:50)	anti-mouse-Ax657 (1:2000)
6	Acetone (10 min; -20°C)	PBS (1X)<br>Tween-20 (0.05%)<br>FBS (5%)	anti-dsDNA-^{127}I (1:50)	anti-mouse-Ax657 (1:2000)
7	Acetone (10 min; -20°C)	PBS (1X)<br>BSA (0.5%)<br>EDTA (5 mM)<br>NaCl (0.5 M)<br>Salmon sperm DNA (200 μg/mL)<br>Human TruStain FcX (20 μg/mL)	anti-dsDNA-^{127}I (1:50)	anti-mouse-Ax657 (1:2000)
8	1.6% PFA (10 min; RT)<br>PBS WASH (x3)<br>Methanol (10 min; -20°C)	PBS (1X)<br>Tween-20 (0.05%)<br>FBS (5%)	anti-dsDNA-^{127}I (1:50)	anti-mouse-Ax657 (1:2000)
9	1.6% PFA (10 min; RT)<br>PBS WASH (x3)<br>Methanol (10 min; -20°C)	PBS (1X)<br>BSA (0.5%)<br>EDTA (5 mM)<br>NaCl (0.5 M)<br>Salmon sperm DNA (200 μg/mL)<br>Human TruStain FcX (20 μg/mL)	anti-dsDNA-^{127}I (1:50)	anti-mouse-Ax657 (1:2000)

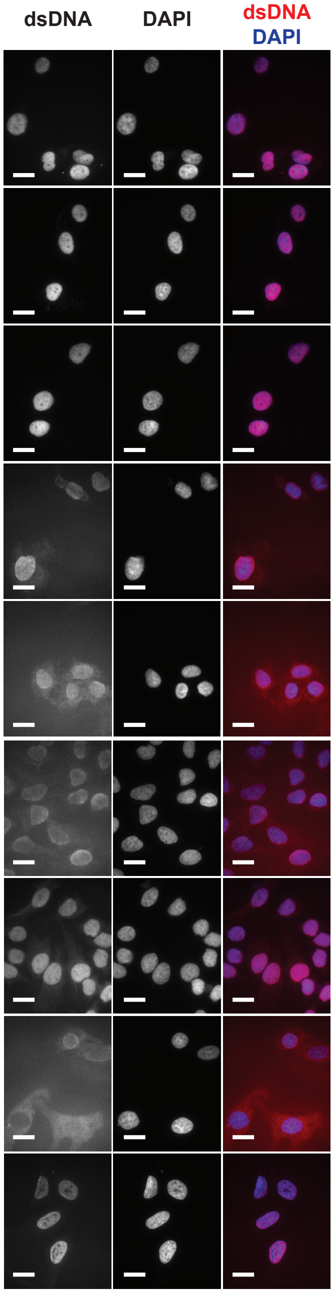

Supplementary Figure 2. Protocol optimization for intracellular staining with MoC-Abs.

HeLa cells were fixed and permeabilized as indicated in the second column. Cells were then blocked for 30 minutes at room temperature as indicated in the third column, stained for 1 hour at room temperature with unconjugated, lanthanide-polymer conjugated (^{162}Dy) or mass-oligonucleotide-conjugated (^{127}I) primary anti-double-stranded DNA (anti-dsDNA), washed three times in PBS, and stained for 1 hour at room temperature with secondary anti-mouse-Alexa 647. Cells were then washed and mounted with fluoromount containing DAPI for confocal microscopy analysis. In HeLa cells stained with anti-dsDNA (sample 1), specific nuclear staining co-localizing with DAPI was observed as expected. HeLa cells stained with anti-dsDNA-^{162}Dy (sample 2) showed the same nuclear staining indicating that that the conjugation process did not alter antibody specificity. The presence of high salt concentration and salmon sperm DNA in cells stained with anti-dsDNA-^{162}Dy (sample 3) did not interfere with antibody staining. HeLa cells fixed and permeabilized with methanol (sample 4), acetone (sample 6), or fixed with 1.6% paraformaldehyde and permeabilized with methanol (sample 8) and stained with anti-dsDNA-^{127}I showed nonspecific cytoplasmic staining. The addition of high salt concentration and salmon sperm DNA in the blocking buffer of HeLa cells fixed and permeabilized with methanol (sample 5) or acetone (sample 7) and stained with anti-dsDNA-^{127}I were stained nonspecifically, although the nonspecific cytoplasmic staining was partially reduced in acetone-treated cells. Fixation with 1.6% paraformaldehyde, permeabilization with methanol, and blocking with a high salt concentration and salmon sperm DNA (sample 9) resulted in a highly specific and background-free visualization of dsDNA in the nucleus of HeLa cells stained with anti-dsDNA-^{127}I. Permeabilization with other reagents such as Triton X-100 or saponin was also compatible with specific MoC-Ab staining (data not shown). Scale bars, 20 μm.

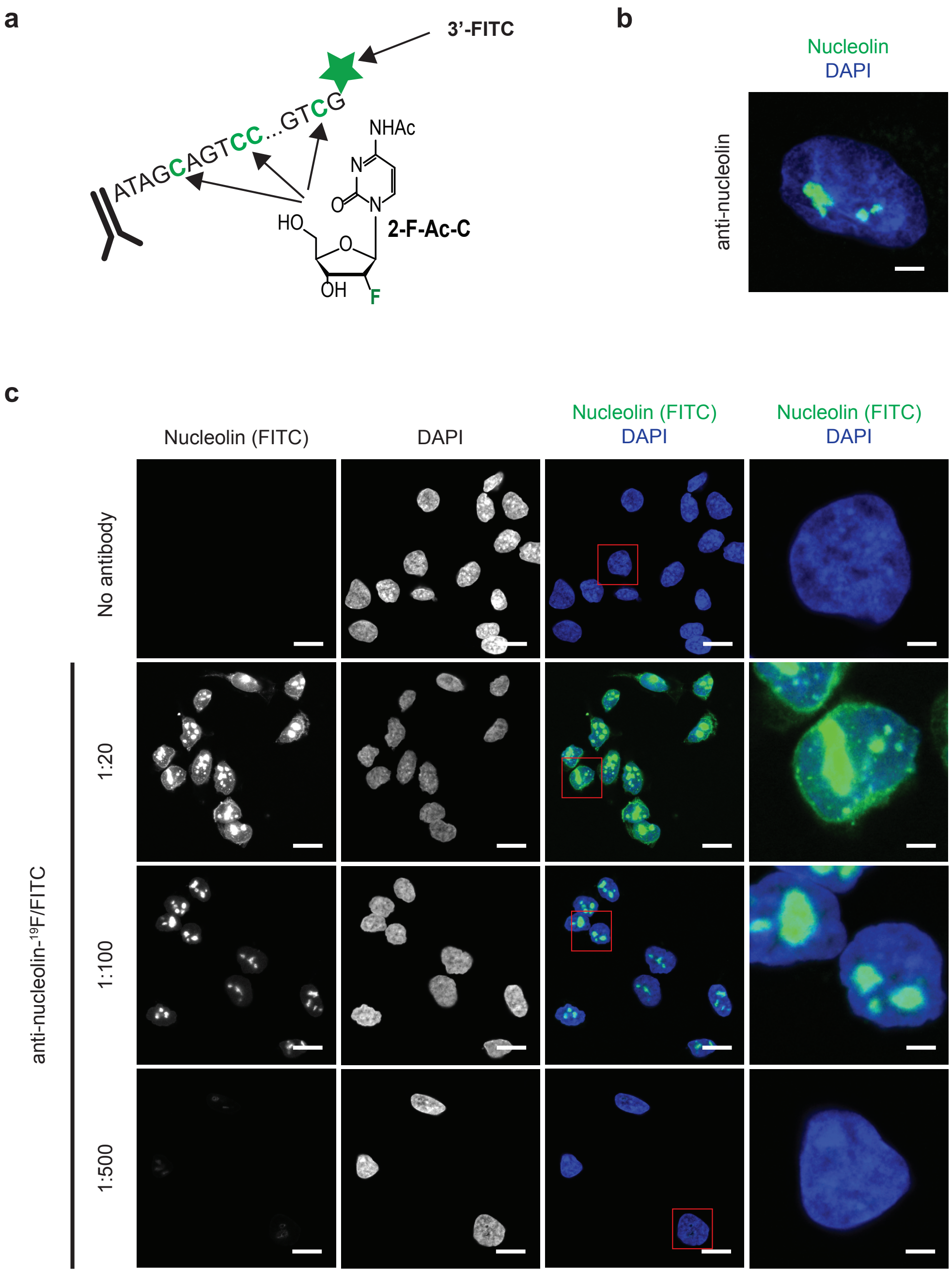
a
3'-FITC
ATAGCAGTCC...GTCG
NHAc
N
N
O
HO
O
OH
F
2-F-Ac-C
b
Nucleolin
DAPI
anti-nucleolin
c
Nucleolin (FITC)
DAPI
Nucleolin (FITC)
DAPI
Nucleolin (FITC)
DAPI
No antibody
1:20
1:100
1:500
anti-nucleolin-19F/FITC

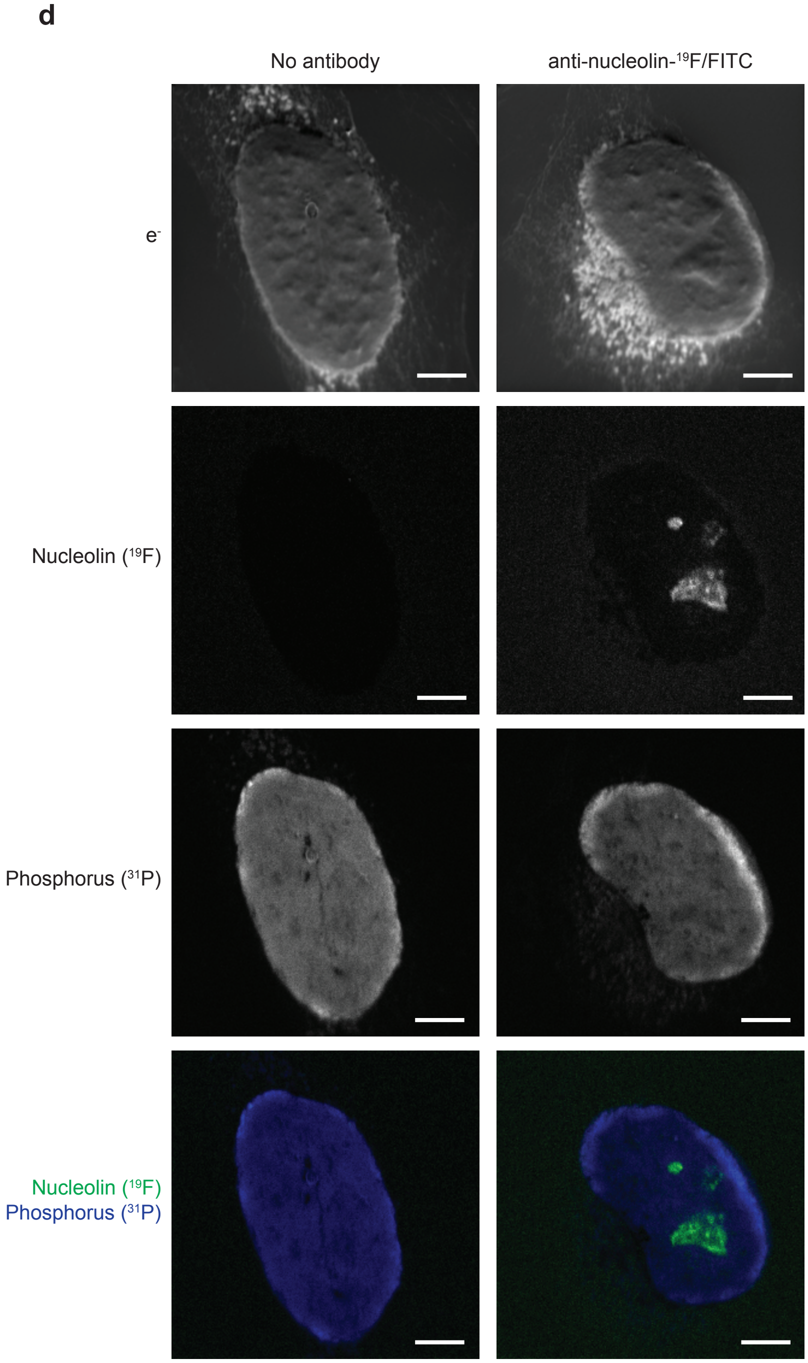
d
No antibody
anti-nucleolin-19F/FITC
e-
Nucleolin (19F)
Phosphorus (31P)
Nucleolin (19F)
Phosphorus (31P)

Supplementary Figure 3. Validation of ^{19}F/FITC MoC-Abs for HD-MIBI.

(**A**) A schematic of the ^{19}F/FITC MoC-Ab. The antibody was conjugated to the 5' end of an oligonucleotide in which all cytidines were replaced with 2-F-Ac-C. The oligonucleotide also had a 3' FITC.

(**B**) Representative confocal microscopy image of a HeLa cell stained with unconjugated anti-nucleolin-^{19}F/FITC and a secondary anti-mouse-Alexa488 (green) and DAPI (blue). Scale bar, 4 μm. n = 1.

(**C**) Representative confocal microscopy images of control HeLa cells (no antibody) or HeLa cells stained with anti-nucleolin-^{19}F/FITC (green) at different concentrations. DAPI is shown in blue. Cells in red boxes in the composite image are shown enlarged in the right column. Scale bars, 20 μm in standard images and 4 μm in enlarged images. A 1:100 dilution of this antibody was used in subsequent experiments. n = 1.

(**D**) Representative HD-MIBI images of a control HeLa cell (no antibody) and HeLa cells stained with anti-nucleolin-^{19}F/FITC. Scale bar, 4 μm. n = 3.

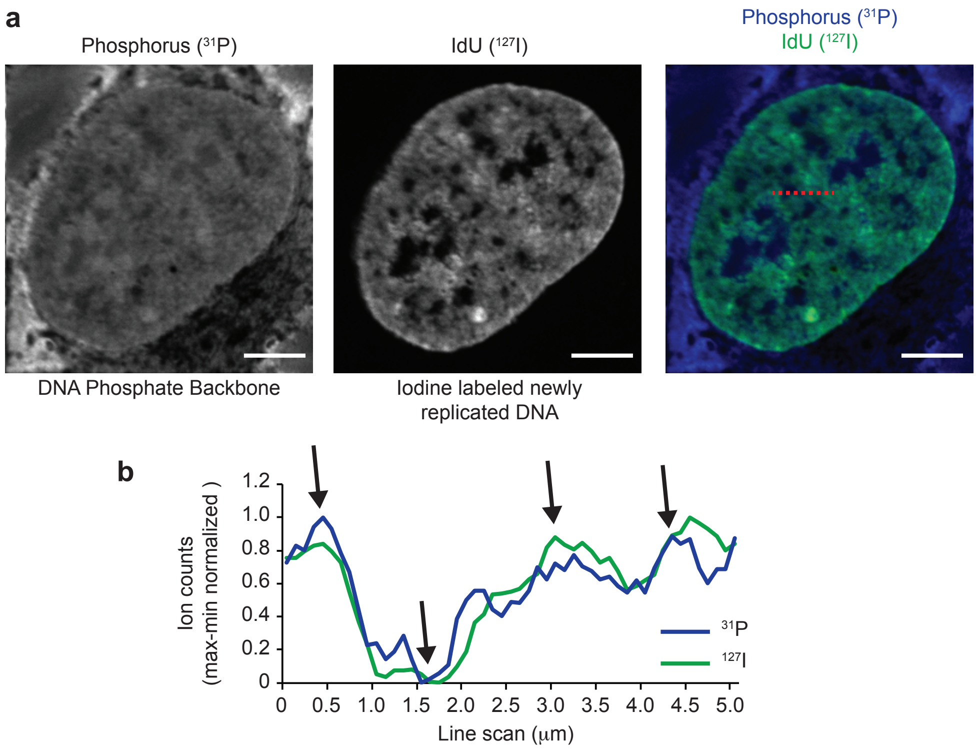

Supplementary Figure 4. High-phosphorus regions within the nucleus mainly originate from the DNA backbone.

(**A**) Representative HD-MIBI image of a HeLa cell treated with 5-iodo-2'-deoxyuridine (IdU) for 24 hours to label DNA. Ion images for phosphorus (^{31}P; left panel) and newly synthesized DNA (^{127}I; middle panel) are overlaid in the right panel. Scale bars, 5 μm. n = 3.

(**B**) Line scan along the dashed red line in the right image in panel A. Raw ion counts were scaled by max-min normalization. Newly replicated DNA (^{127}I; green line) and phosphorus (^{31}P; blue line) in the nucleus show a similar pattern, confirming that high-phosphorus regions mainly originate from the DNA backbone rather than from RNA or phosphorylated proteins.

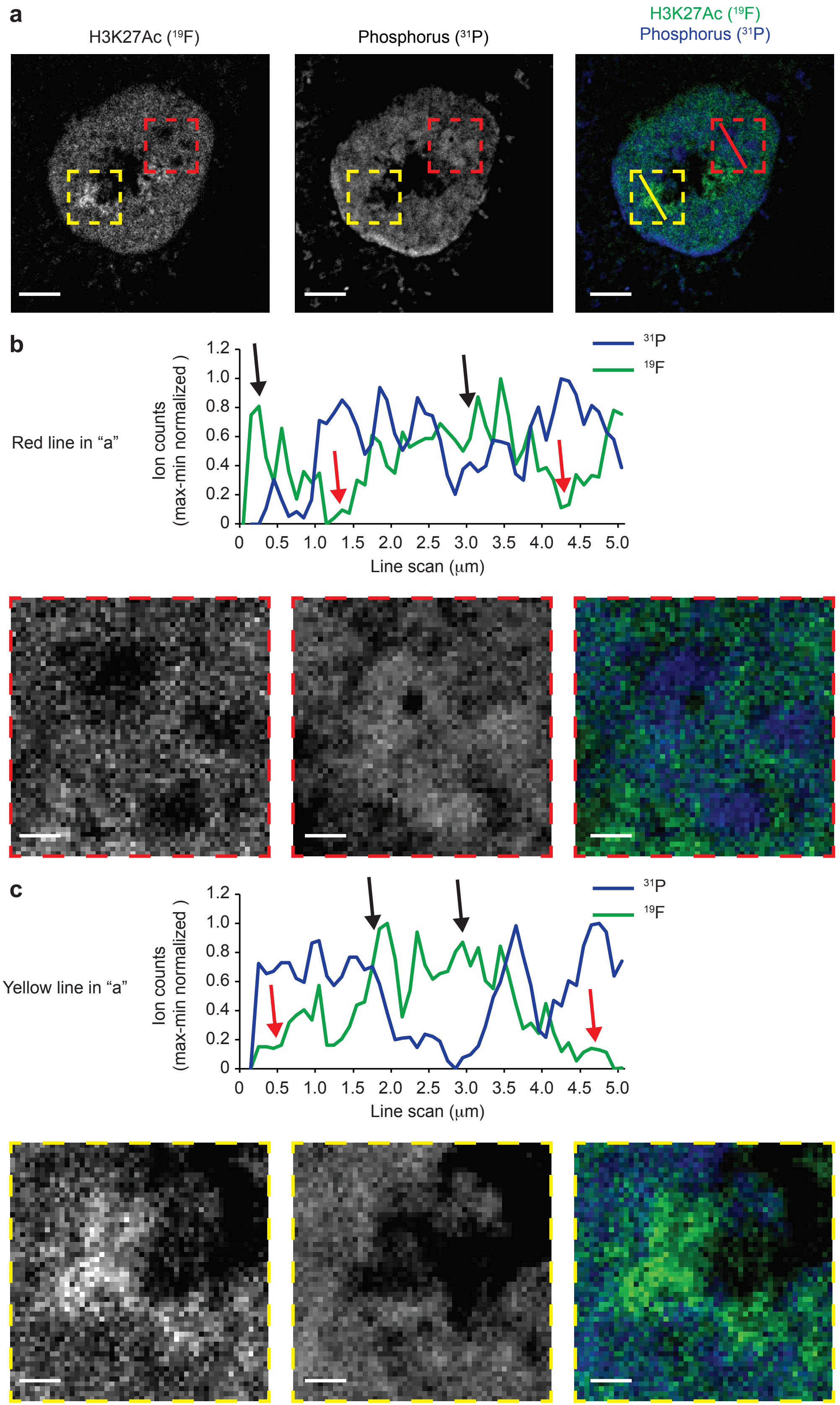

a
H3K27Ac (19F)
Phosphorus (31P)
H3K27Ac (19F)
Phosphorus (31P)
b
Red line in "a"
Ion counts (max-min normalized)
1.2 1.0 0.8 0.6 0.4 0.2 0
0 0.5 1.0 1.5 2.0 2.5 3.0 3.5 4.0 4.5 5.0
Line scan (μm)
31P
19F
c
Yellow line in "a"
Ion counts (max-min normalized)
1.2 1.0 0.8 0.6 0.4 0.2 0
0 0.5 1.0 1.5 2.0 2.5 3.0 3.5 4.0 4.5 5.0
Line scan (μm)
31P
19F

Supplementary Figure 5. Regions of active transcription within the nucleus mainly originate from low-phosphorus regions.

(**A**) Representative HD-MIBI image of a HeLa cell stained with anti-H3K27Ac-^{19}F/FITC. Ion images for H3K27Ac (^{19}F; left panel) and phosphorus (^{31}P; middle panel) are overlaid in the right panel. Scale bars, 4 µm. Cell in the composite image is also shown in Fig. 1D. n = 3.

(**B**-**C**) Line scan along the lines in the boxes outlined in **B**) red and **C**) yellow dashed lines in the image to the right in panel A. Raw ion counts were scaled by mix-man normalization. Black arrows point to regions of high H3K27Ac counts (green line) that are anti-correlated with phosphorus counts (blue line). Red arrows point to low H3K27Ac counts (green line) that are anti-correlated with phosphorus counts (blue line). Images below the line scans are magnified from regions boxed in panel A. Scale bars, 800 nm.

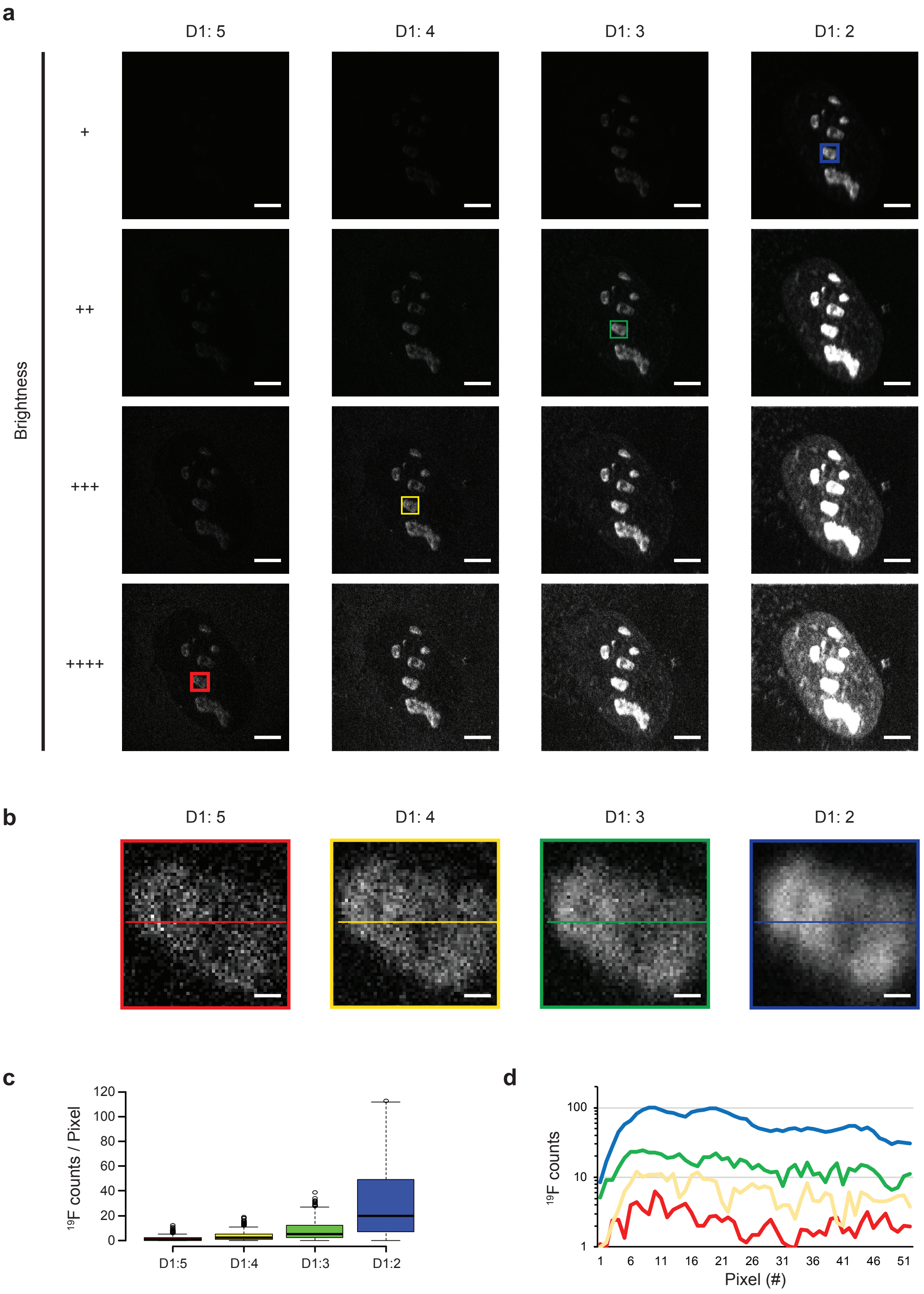

a
D1: 5
D1: 4
D1: 3
D1: 2
Brightness
+
++
+++
++++
b
D1: 5
D1: 4
D1: 3
D1: 2
c
¹⁹F counts / Pixel
0
20
40
60
80
100
120
D1:5
D1:4
D1:3
D1:2
d
¹⁹F counts
1
10
100
1
6
11
16
21
26
31
36
41
46
51
Pixel (#)

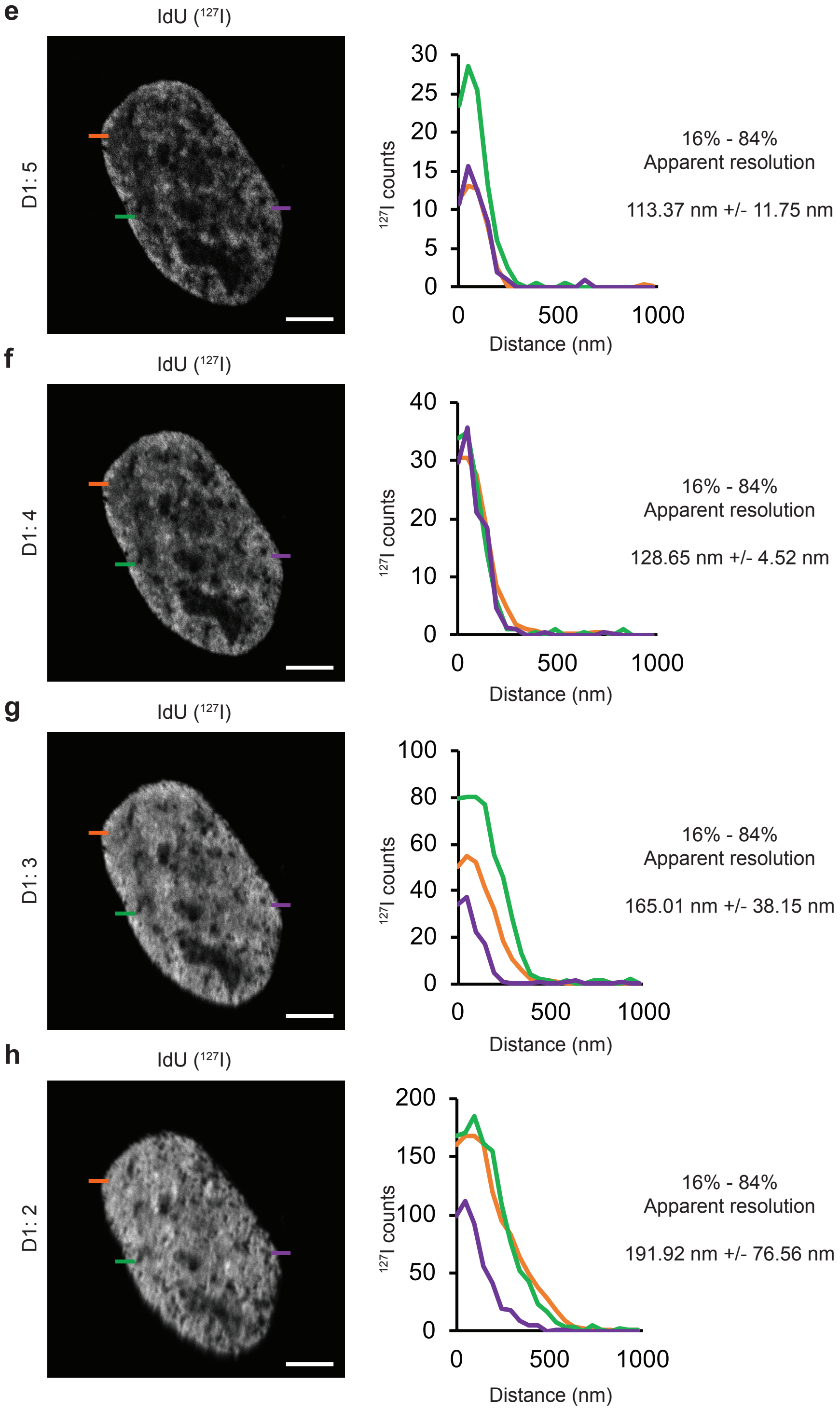
e
IdU (127I)
D1: 5
16% - 84%
Apparent resolution
113.37 nm +/- 11.75 nm
f
IdU (127I)
D1: 4
16% - 84%
Apparent resolution
128.65 nm +/- 4.52 nm
g
IdU (127I)
D1: 3
16% - 84%
Apparent resolution
165.01 nm +/- 38.15 nm
h
IdU (127I)
D1: 2
16% - 84%
Apparent resolution
191.92 nm +/- 76.56 nm
127I counts
Distance (nm)

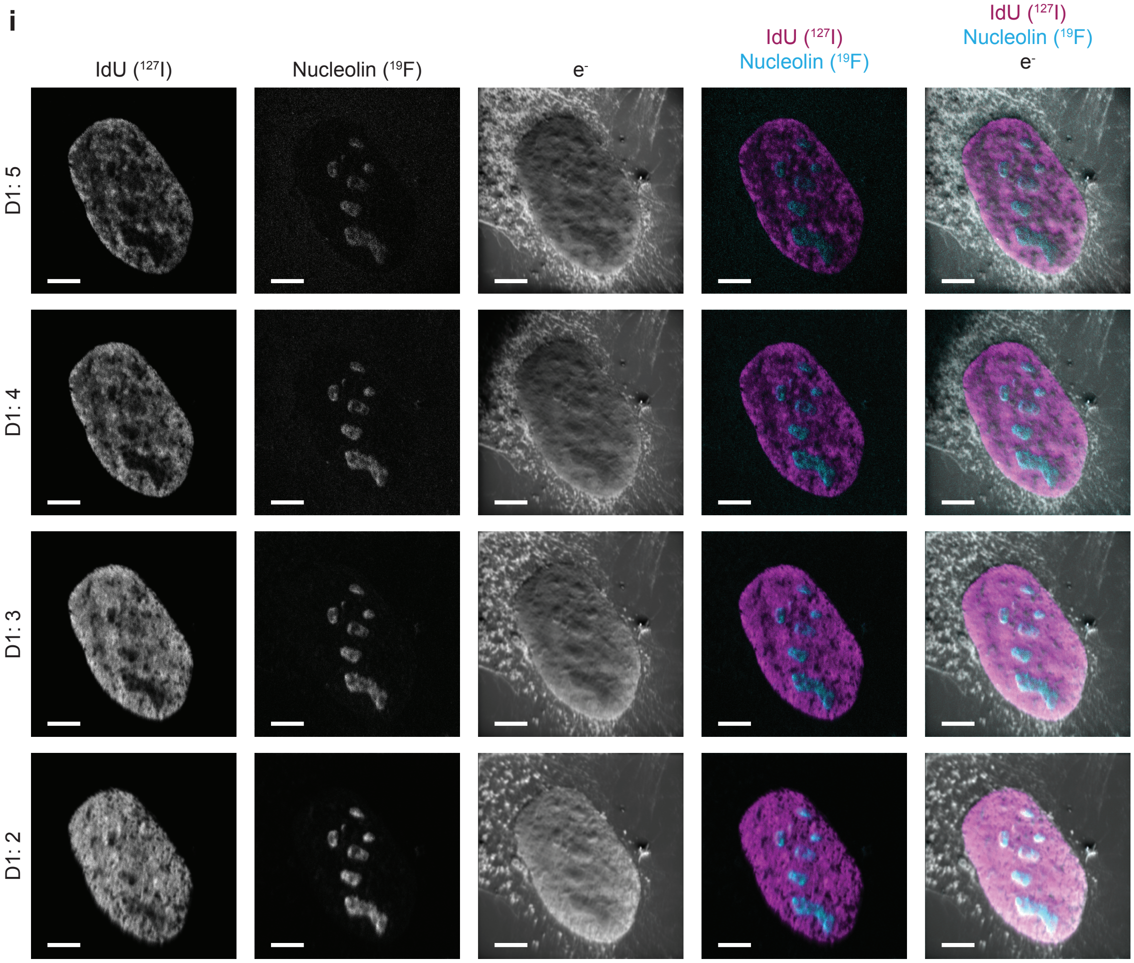

Supplementary Figure 6. Beam current scales with ion counts per pixel at the expense of resolution.

HeLa cells were labeled with IdU for 24 hours and stained with anti-nucleolin-^{19}F/FITC. The images were acquired with a constant 25 * 25 µm field of view, 1 ms dwell time per pixel, and 512 * 512 pixels (xy). The beam current was increased every 10 planes by changing the D1 aperture width to obtain a beam diameter of ~50 nm (D1: 5), ~75 nm (D1: 4), ~100 nm (D1: 3) and ~150 nm (D1: 2), with a current of ~0.7 pA (D1: 5), ~1.2 pA (D1: 4), ~3 pA (D1: 3) and ~6 pA (D1: 2). n = 1.

(**A**) Representative HD-MIBI images of nucleolin staining in the same HeLa cell using different aperture widths. Different brightness intensities to normalize for variation in ion counts are shown for clarity. Scale bars, 4 μm.

(**B**) Enlarged images of colored boxes within images in panel A show an individual nucleolus. Details are increasingly lost as the beam current is elevated. Scale bars, 400 nm.

(**C**) Ion count per pixel of all pixels in images shown in panel B. Each colored boxplot represents color-coded images in panel B. Boxplots from samples analyzed at higher currents (D1: 2 and 3) show higher ion counts per pixel than boxplots from samples analyzed at lower currents (D1: 4 and 5), highlighting loss of ion counts per pixel at lower current. The center of the box corresponds to the median. The minima and maxima bound of box correspond to the 25th and 75th percentiles, respectively. The whiskers extend from the minima and maxima bounds of box to the largest value no further than 1.5 times the inter-quartile range. The outliers are shown as dots. N = 3421 pixels per condition.

(**D**) Line scans from lines within images shown in panel B. Line scans at high current (D1: 2 and 3) are smoother than line scans at low current (D1: 4 and 5), showing loss of resolution at higher current.

(**E-H**) Quantification of lateral resolution using a beam diameter of **E**) ~50 nm (D1: 5), **F**) ~75 nm (D1: 4), **G**) ~100 nm (D1: 3), and **H**) ~150 nm (D1: 2). (Left) Representative HD-MIBI images of newly replicated DNA (IdU) staining in the same HeLa cell. Scale bars, 4 μm. (Right) Line scan for each colored bar in the images shown on the left. The lateral resolution calculated using the 16%-84% criterion is shown as an average of each of the three analyzed lines ± s.d.

(**I**) Single channel and composite images of images shown in Fig. 1E of $^{-}$e, nucleolin, and IdU.

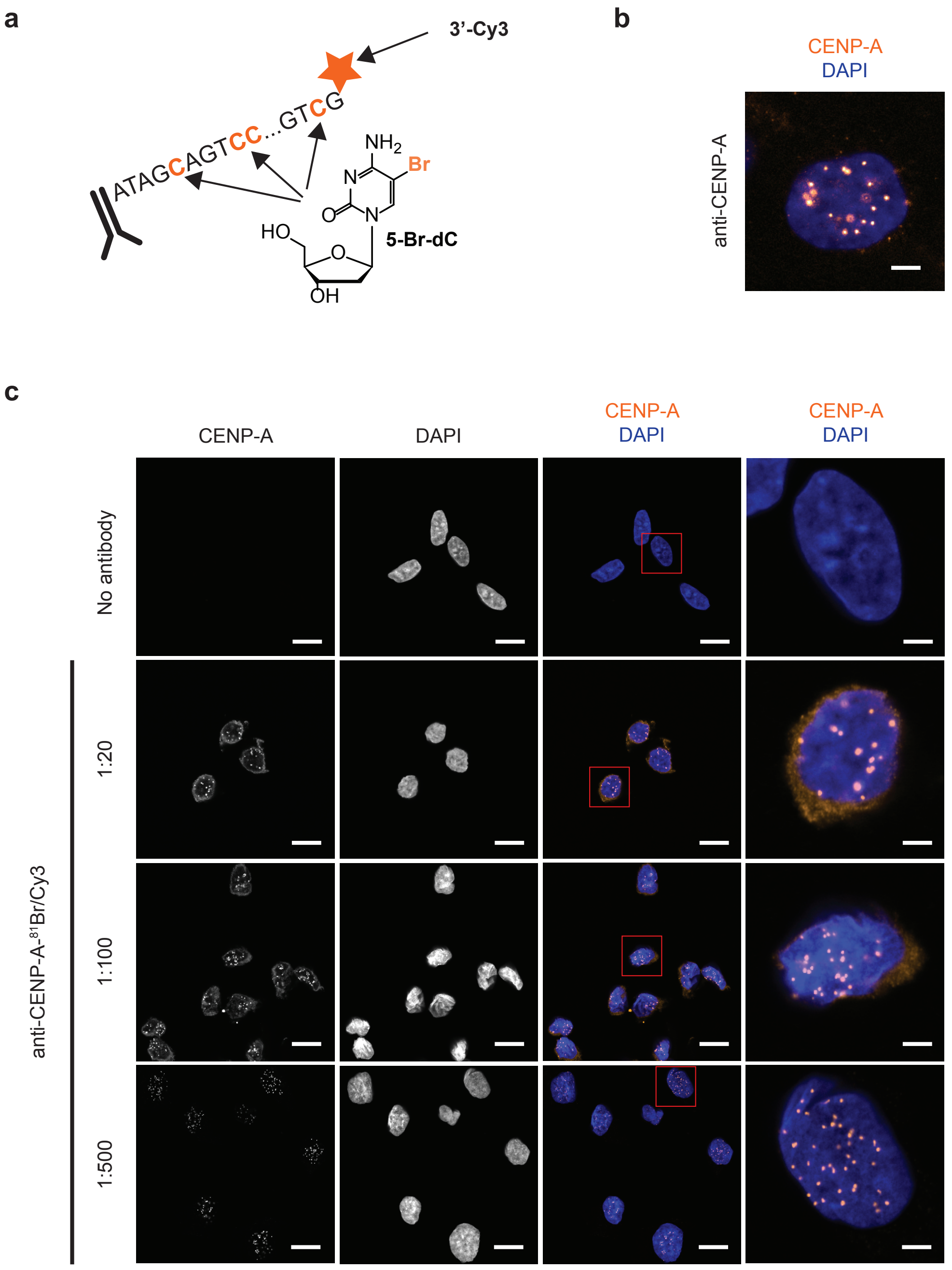
a
3'-Cy3
ATAGCAGTCC...GTCG
NH2
Br
N
O
N
HO
O
OH
5-Br-dC
b
CENP-A
DAPI
anti-CENP-A
c
CENP-A
DAPI
CENP-A
DAPI
CENP-A
DAPI
No antibody
1:20
1:100
1:500
anti-CENP-A-81Br/Cy3

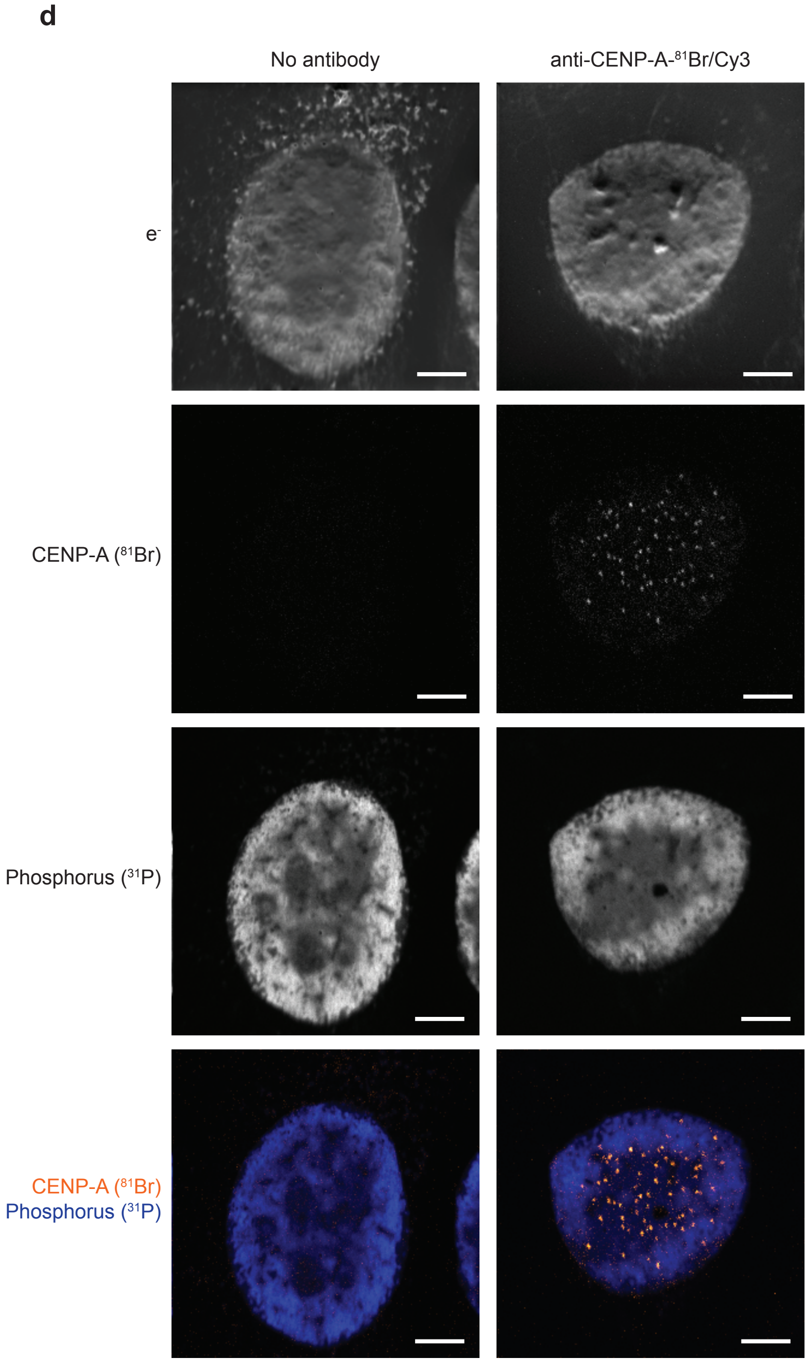
d
No antibody
anti-CENP-A-81Br/Cy3
e-
CENP-A (81Br)
Phosphorus (31P)
CENP-A (81Br)
Phosphorus (31P)

Supplementary Figure 7. Validation of ^{81}Br/Cy3 MoC-Abs for HD-MIBI.

(**A**) A schematic of the ^{81}Br/Cy3 MoC-Ab. The antibody was conjugated to the 5’ end of an oligonucleotide in which all cytidines were replaced by 5-Br-dC. The oligonucleotide also had a 3’ Cy3.

(**B**) Representative confocal microscopy image of a HeLa cell stained with unconjugated anti-CENP-A-^{81}Br/Cy3 and a secondary anti-mouse-Alexa488 (orange) and DAPI (blue). Scale bar, 4 µm. n = 1.

(**C**) Representative confocal microscopy images of control HeLa cells (no antibody) and HeLa cells stained with anti-CENP-A-^{81}Br/Cy3 (orange) at three different concentrations. DAPI is shown in blue. Cells in red boxes in the composite image are enlarged in the right column. Scale bars, 20 µm in standard images and 4 µm in enlarged images. A 1:500 dilution of this antibody was used in subsequent experiments. n = 1.

(**D**) Representative HD-MIBI images of control HeLa cell (no antibody) and HeLa cell stained with anti-CENP-A-^{81}Br/Cy3. Scale bar, 4 µm. n = 3.

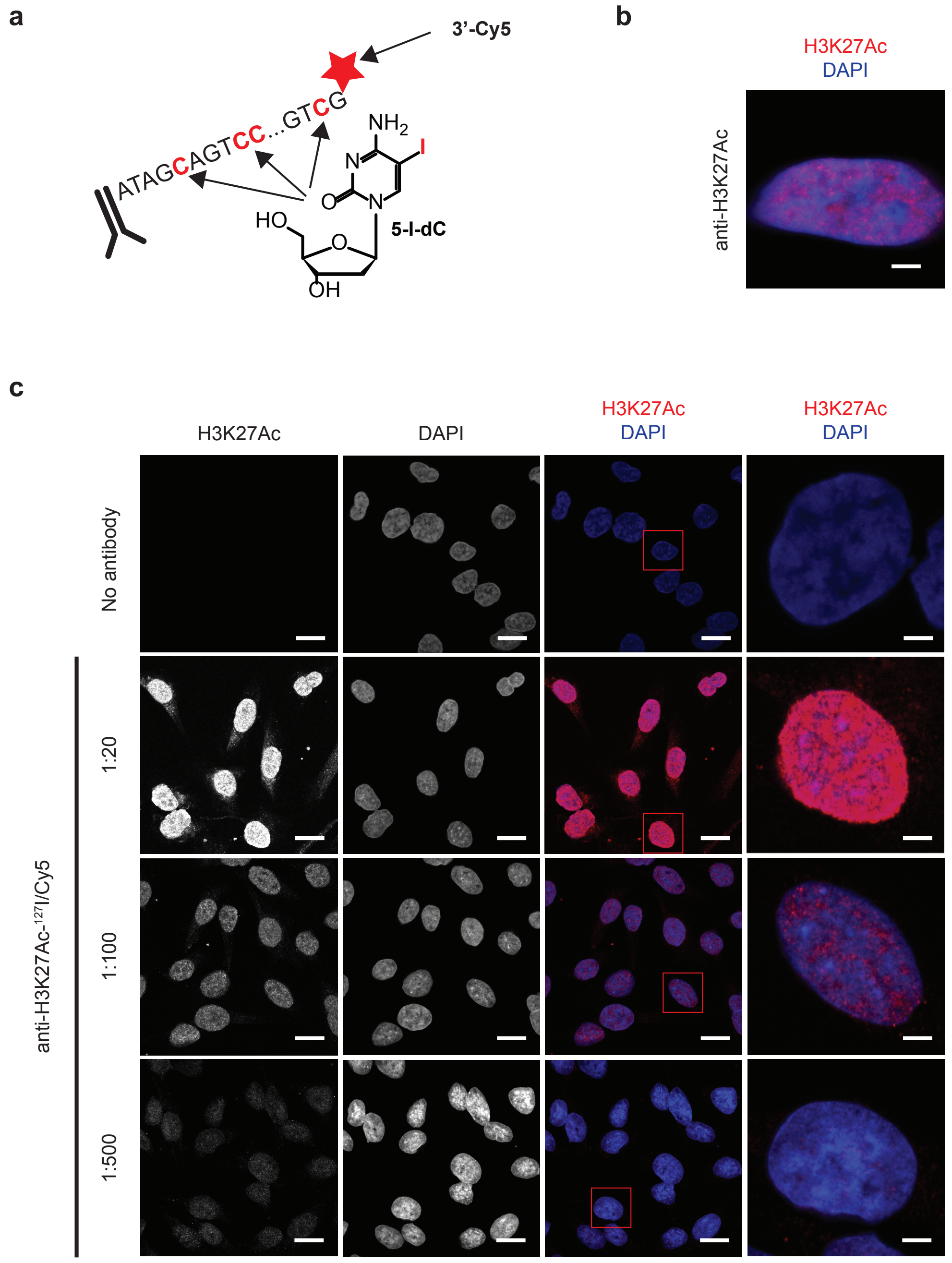
a
3'-Cy5
ATAGCAGTCC...GTCG
NH2
I
N
O
N
HO
O
OH
5-I-dC
b
H3K27Ac
DAPI
anti-H3K27Ac
c
H3K27Ac
DAPI
H3K27Ac
DAPI
H3K27Ac
DAPI
No antibody
1:20
1:100
1:500
anti-H3K27Ac-127I/Cy5

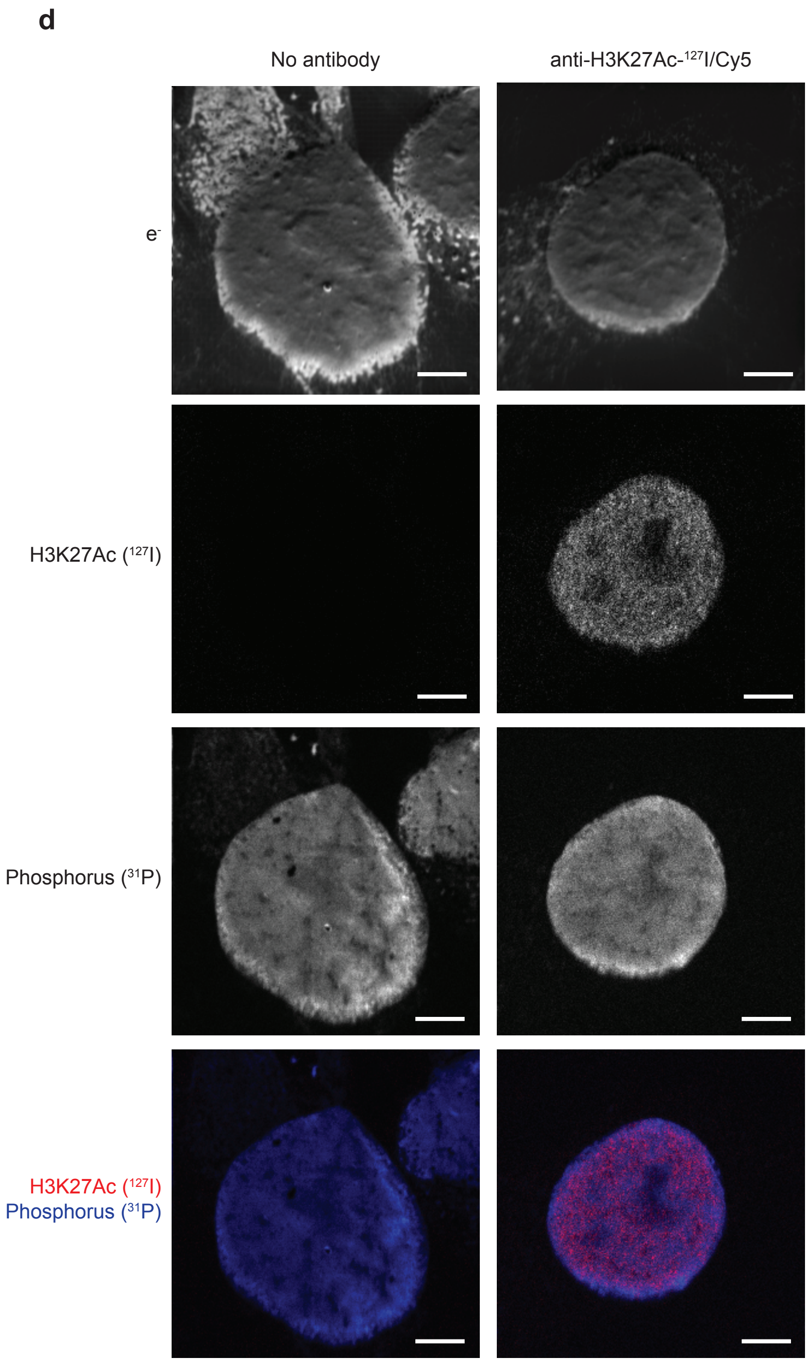

d
No antibody
anti-H3K27Ac-127I/Cy5
e-
H3K27Ac (127I)
Phosphorus (31P)
H3K27Ac (127I)
Phosphorus (31P)

Supplementary Figure 8. Validation of ^{127}I/Cy5 MoC-Abs for HD-MIBI.

(**A**) A schematic of the ^{127}I/Cy5 MoC-Ab. The antibody was conjugated to the 5' end of an oligonucleotide in which all cytidines were replaced by 5-I-dC. The oligonucleotide also had a 3' Cy5.

(**B**) Representative confocal microscopy image of a HeLa cell stained with unconjugated anti-H3K27Ac and a secondary anti-rabbit-Alexa647 (red) and DAPI (blue). Scale bar, 4 µm. n = 1.

(**C**) Representative confocal microscopy images of control HeLa cells (no antibody) and HeLa cells stained with anti-H3K27Ac-^{127}I/Cy5 (red) at three different concentrations. DAPI is shown in blue. Cells in red boxes in the composite image are enlarged in the right column. Scale bars, 20 µm in standard images and 4 µm in enlarged images. A 1:100 dilution of this antibody was used in subsequent experiments. n = 1.

(**D**) Representative HD-MIBI images of control HeLa cell (no antibody) and HeLa cell stained with anti-H3K27Ac-^{127}I/Cy5. Scale bar, 4 µm. n = 3.

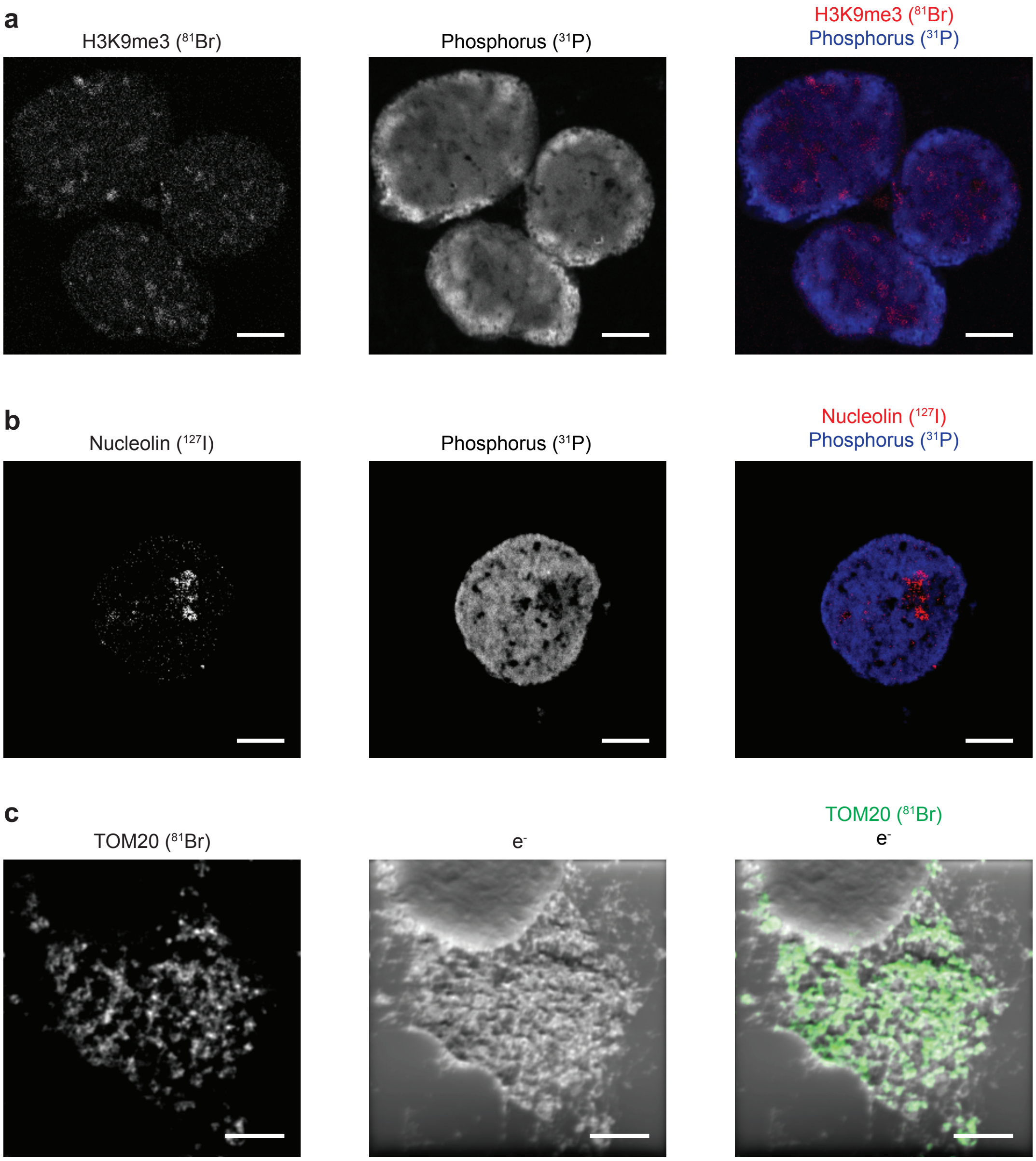

Supplementary Figure 9. Validation of MoC-Abs containing 5-Br-dC or 5-I-dC for HD-MIBI by targeting other proteins.

(**A**) Representative HD-MIBI image of a HeLa cell stained with anti-H3K9me3-^{81}Br/Cy3 (red). Phosphorus is shown in blue. Scale bar, 4 μm. n = 3.

(**B**) Representative HD-MIBI image of a HeLa cell stained with anti-nucleolin-^{127}I/Cy5 (red). Phosphorus is shown in blue. Scale bar, 4 μm. n = 3.

(**C**) Representative HD-MIBI images of a HeLa cell stained with anti-TOM20-^{81}Br/Cy3 (green). Scale bar, 4 µm. n = 1.

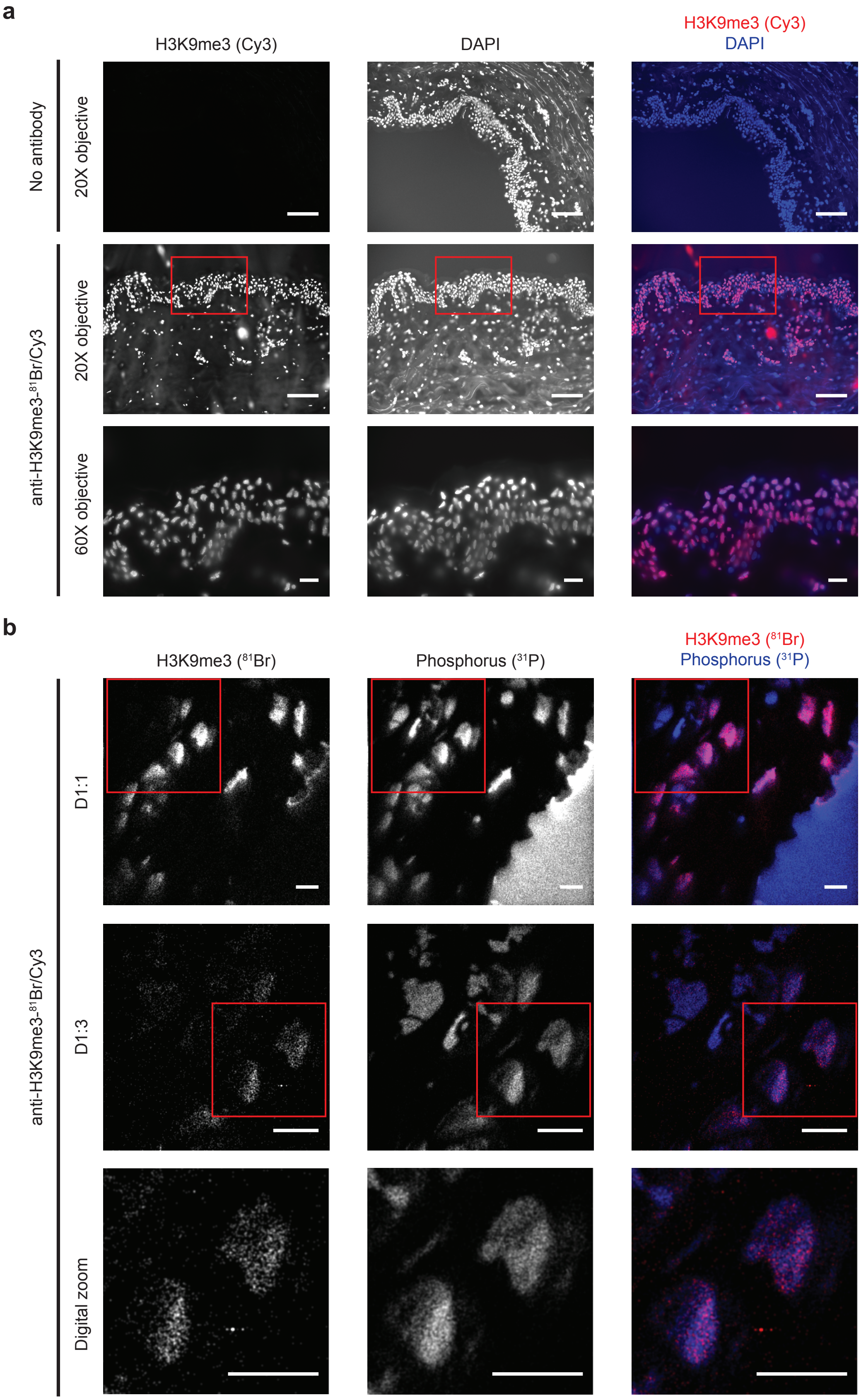
a
H3K9me3 (Cy3)
DAPI
H3K9me3 (Cy3)
DAPI
No antibody
20X objective
anti-H3K9me3-81Br/Cy3
20X objective
60X objective
b
H3K9me3 (81Br)
Phosphorus (31P)
H3K9me3 (81Br)
Phosphorus (31P)
anti-H3K9me3-81Br/Cy3
D1:1
D1:3
Digital zoom

Supplementary Figure 10. Validation of MoC-Abs in frozen tissue sections.

(**A**) Representative confocal microscopy images of control human adult skin section (no antibody) and human adult skin section stained with anti-H3K9me3-^{81}Br/Cy3 (red) at two magnifications. DAPI is shown in blue. Fields of view in red boxes in the 20x images were re-captured with a 60x objective. Scale bars, 100 µm in 20X images and 20 µm in 60X images. n = 1.

(**B**) Representative HD-MIBI images of the epidermis of a human adult skin section stained with anti-H3K9me3-^{81}Br/Cy3. Scale bar, 4 µm. n = 1.

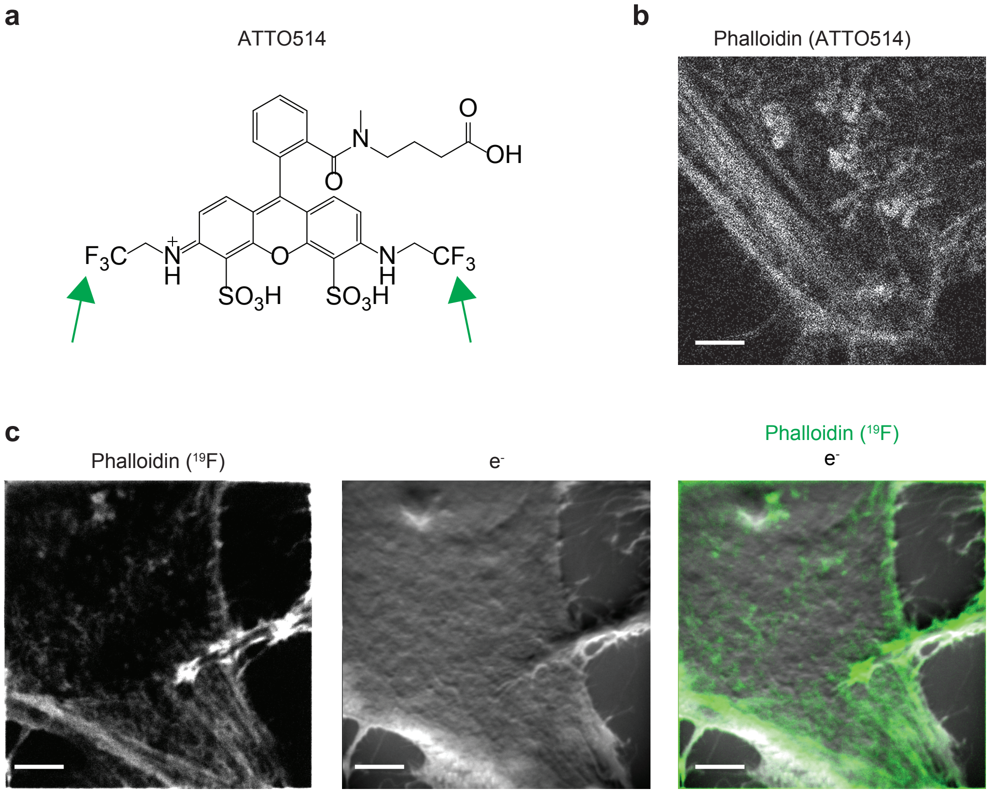

Supplementary Figure 11. Validation of Phalloidin-ATTO514 for HD-MIBI.

(**A**) Chemical structure of ATTO 514. Green arrows indicate the presence of the six ^{19}F atoms in ATTO 514.

(**B**) Representative confocal microscopy image of a HeLa cell stained with phalloidin-ATTO514. Scale bar, 4 µm. n = 1.

(**C**) Representative HD-MIBI images of a HeLa cell stained with phalloidin-ATTO514. Scale bar, 4 µm. n = 1.

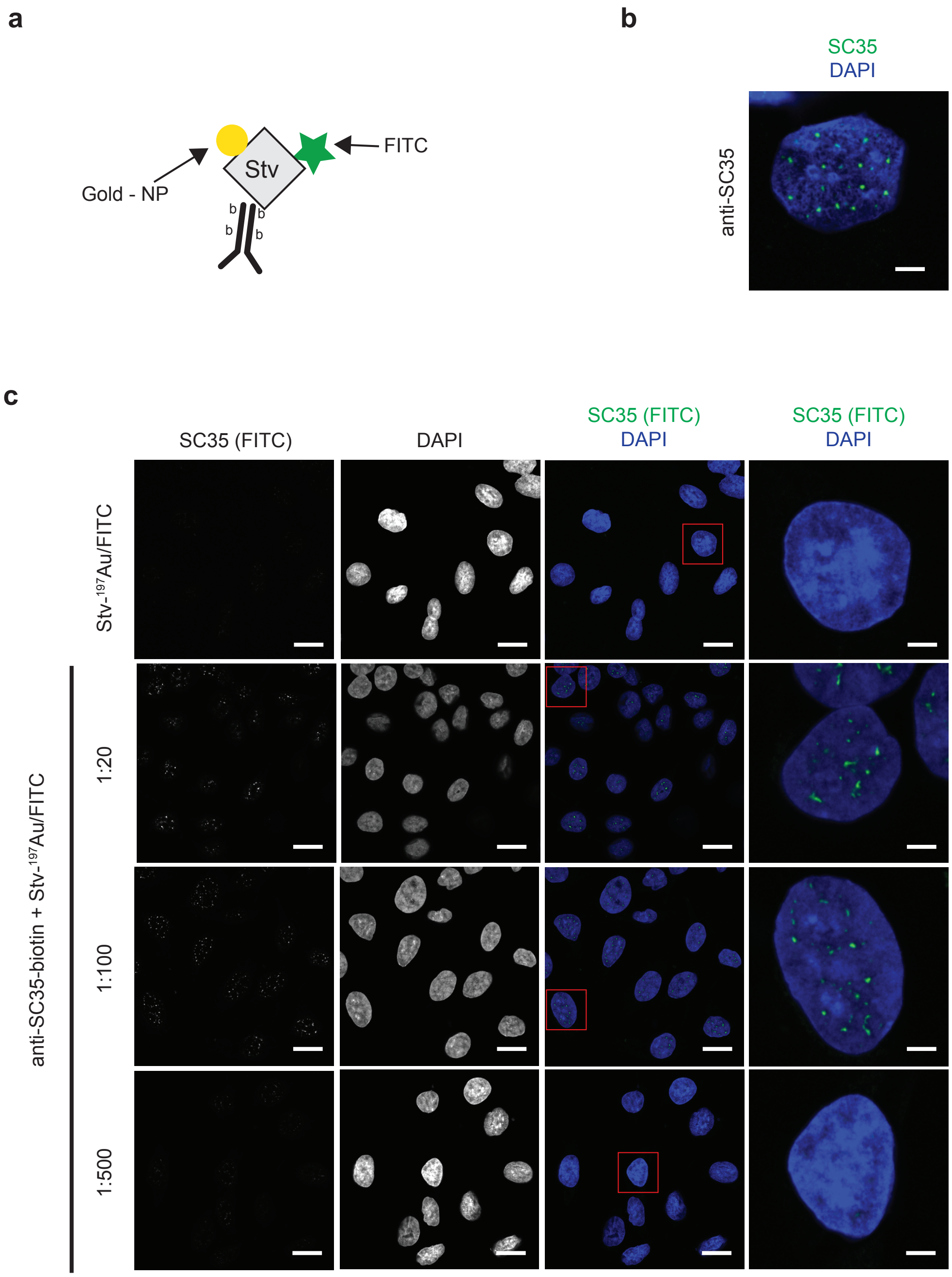
a
Stv
FITC
Gold - NP
b
b
b
b
b
SC35
DAPI
anti-SC35
c
SC35 (FITC)
DAPI
SC35 (FITC)
DAPI
SC35 (FITC)
DAPI
Stv-197Au/FITC
anti-SC35-biotin + Stv-197Au/FITC
1:20
1:100
1:500

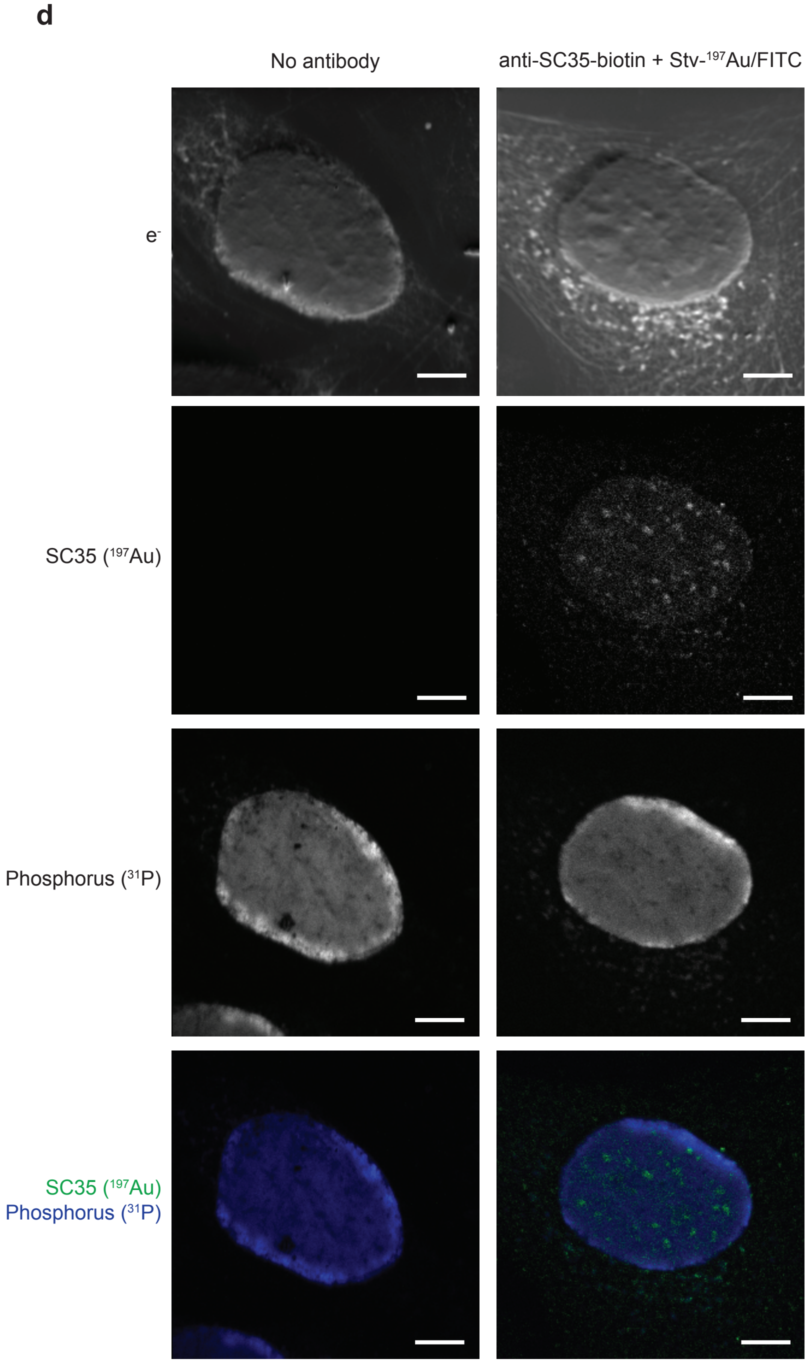
d
No antibody
anti-SC35-biotin + Stv-197Au/FITC
e-
SC35 (197Au)
Phosphorus (31P)
SC35 (197Au)
Phosphorus (31P)

Supplementary Figure 12. Validation of biotin-conjugated antibodies stained with ^{197}Au/FITC-conjugated streptavidin.

(**A**) A schematic of the antibody conjugation strategy. Primary antibodies were conjugated to biotin and subsequently stained with ^{197}Au/FITC-conjugated streptavidin. The ^{197}Au consists of 1.4 nm nanoparticles, a diameter below the theoretical HD-MIBI resolution limit.

(**B**) Representative confocal microscopy image of a HeLa cell stained with unconjugated anti-SC35 and a secondary anti-mouse-Alexa488 (green). DAPI (blue) was used to stain the nucleus. Scale bars, 4 µm. n = 1.

(**C**) Representative confocal microscopy images of HeLa cells stained with streptavidin-^{197}Au/FITC and HeLa cells stained with anti-SC35-biotin followed by streptavidin-^{197}Au/FITC at different concentrations of the primary antibody and 1:40 of streptavidin-^{197}Au/FITC (green). DAPI (blue) was used to stain nuclei. Scale bars, 20 µm. Cells in red boxes in the composite image are magnified in the right column. Scale bars, 4 µm. A 1:100 dilution of this antibody was used on following experiments. n = 1.

(**D**) Representative HD-MIBI image of a control HeLa cell (no antibody) and a HeLa cell stained with anti-SC35-biotin followed by streptavidin-^{197}Au/FITC. Scale bar, 4 µm. n = 3.

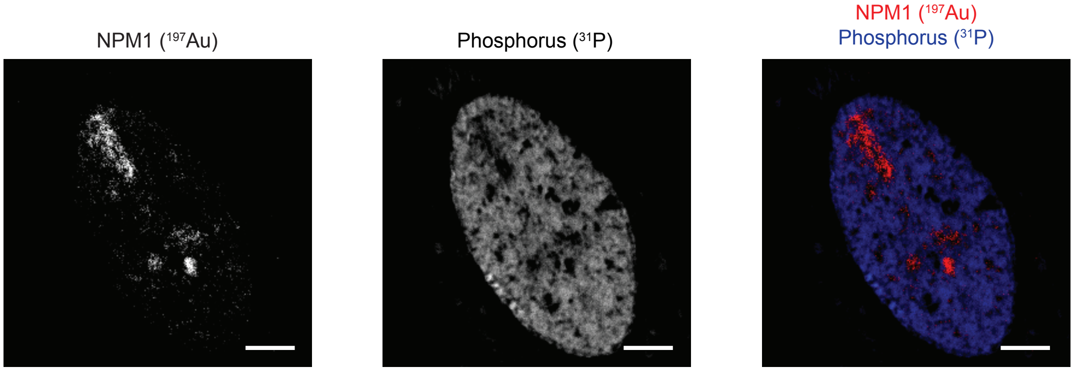

Supplementary Figure 13. Validation of biotin-conjugated antibodies stained with ^{197}Au/FITC-conjugated streptavidin for HD-MIBI by targeting other proteins.

Representative HD-MIBI image of a HeLa cell stained with anti-NPM1-biotin followed by streptavidin-^{197}Au/FITC (red). Phosphorus is shown in blue. Scale bars, 4 µm. n = 2.

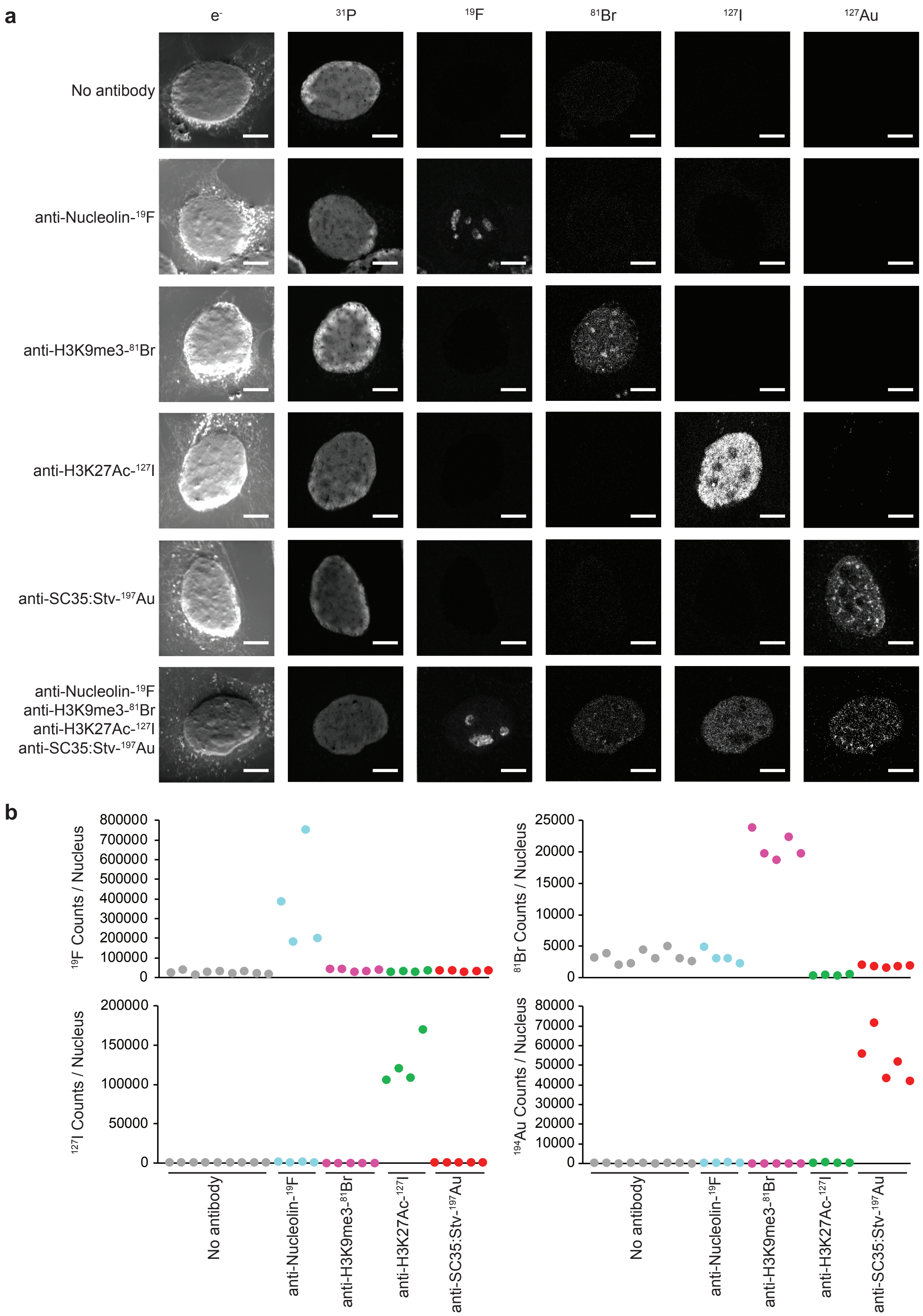
a
e-
31P
19F
81Br
127I
127Au
No antibody
anti-Nucleolin-19F
anti-H3K9me3-81Br
anti-H3K27Ac-127I
anti-SC35:Stv-197Au
anti-Nucleolin-19F
anti-H3K9me3-81Br
anti-H3K27Ac-127I
anti-SC35:Stv-197Au
b
19F Counts / Nucleus
800000
700000
600000
500000
400000
300000
200000
100000
0
81Br Counts / Nucleus
25000
20000
15000
10000
5000
0
127I Counts / Nucleus
200000
150000
100000
50000
0
194Au Counts / Nucleus
80000
70000
60000
50000
40000
30000
20000
10000
0
No antibody
anti-Nucleolin-19F
anti-H3K9me3-81Br
anti-H3K27Ac-127I
anti-SC35:Stv-197Au
No antibody
anti-Nucleolin-19F
anti-H3K9me3-81Br
anti-H3K27Ac-127I
anti-SC35:Stv-197Au

Supplementary Figure 14. Assessment of HD-MIBI channel crosstalk.

(**A**) Representative HD-MIBI images of a control HeLa cell control (no antibody) and HeLa cells stained with the indicated antibodies. Each row represents a staining condition and each column the ion channel extracted for the indicated isotope. Scale bars, 4 µm.

(**B**) Quantification of channel crosstalk. Unstained HeLa cell (n = 9) and HeLa cell stained with anti-nucleolin-^{19}F/FITC (n = 4), anti-H3K9me3-^{81}Br/Cy3 (n= 5), anti-H3K27Ac-^{127}I/Cy5 (n = 4), and anti-SC35-biotin (recognized by streptavidin-^{197}Au/FITC; n = 5) were analyzed by HD-MIBI. A mask was created for each cell using the phosphorus channel and the total ^{19}F, ^{81}Br, ^{127}I and ^{197}Au ion counts of 10 consecutive scans per nucleus were extracted and plotted.

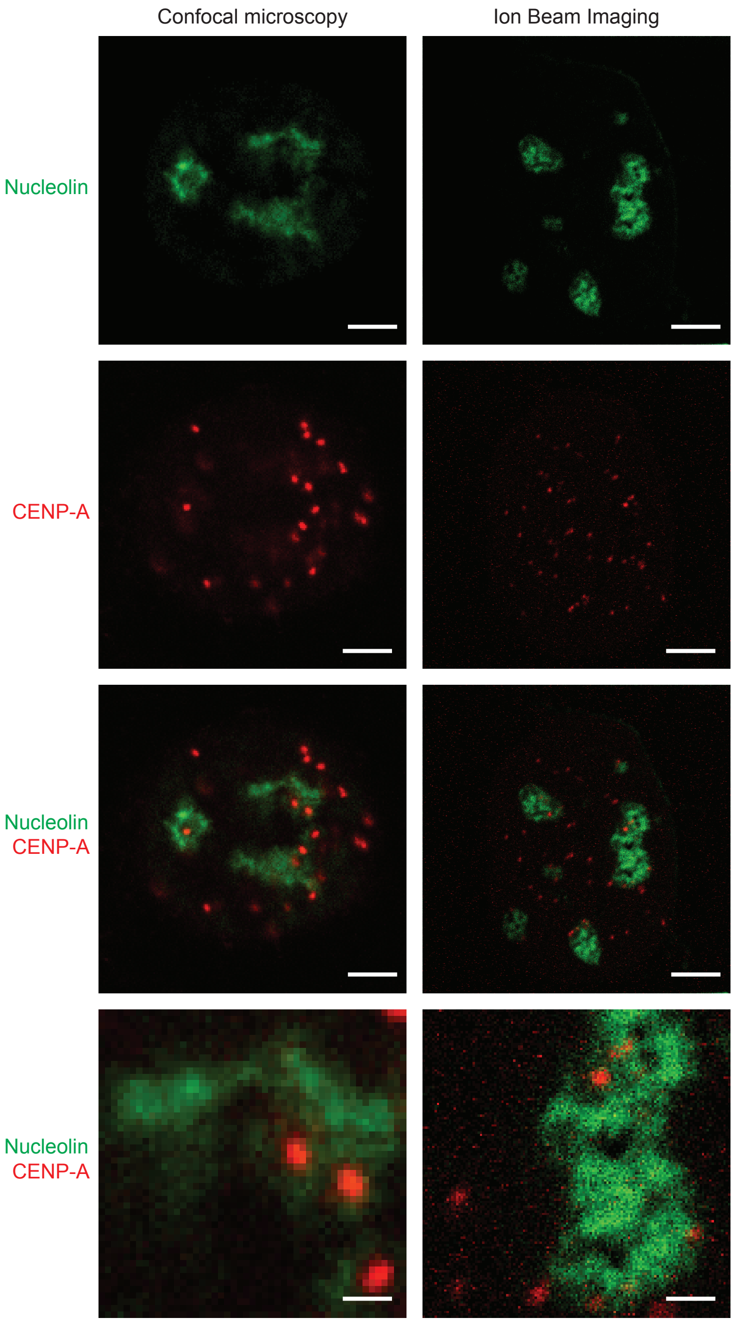

Supplementary Figure 15. Two-channel HD-MIBI on HeLa cells using a beam diameter of ~50 nm (D1: 5). Representative confocal microscopy (left) and HD-MIBI (right) images of HeLa cells stained with anti-nucleolin-^{19}F/FITC and anti-CENP-A-^{81}Br/Cy3 MoC-Ab. Scale bars, 4 µm (top 3 rows) and 1 µm (bottom row). n = 1.

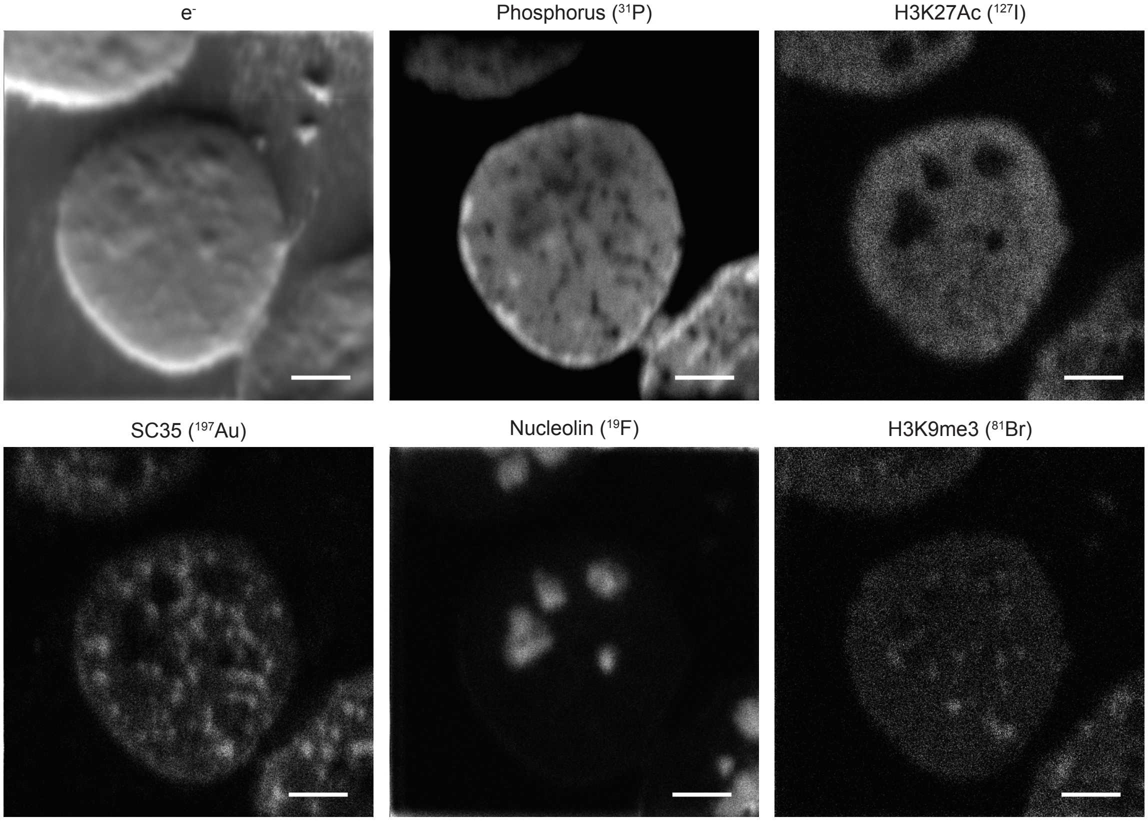

Supplementary Figure 16. Six-channel HD-MIBI on HeLa cells using a beam diameter of ~50 nm (D1: 5). Representative HD-MIBI image of a HeLa cell stained with anti-nucleolin-^{19}F/FITC, anti-H3K9me3-^{81}Br/Cy3, anti-H3K27Ac-^{127}I/Cy5, and anti-SC35-biotin (recognized by streptavidin-^{197}Au/FITC). Images of the e^{-}, nucleolin (^{19}F), DNA (^{31}P), H3K9me3 (^{81}Br), H3K27Ac (^{127}I), and SC35 (^{197}Au) were simultaneously acquired. A composite image is shown in Fig. 2D. Scale bars, 5 µm. n = 2.

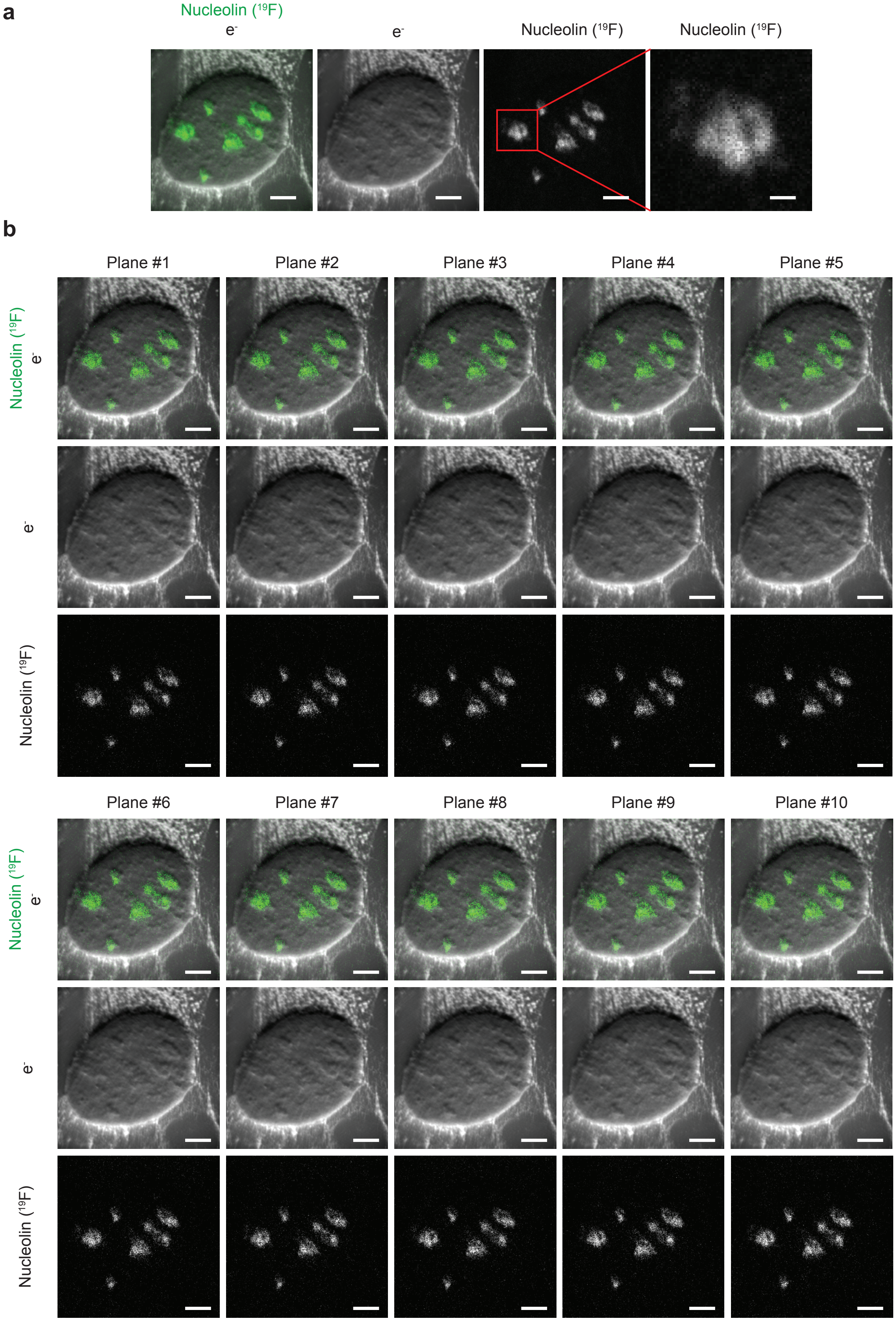
a
Nucleolin (^{19}F)
e⁻
e⁻
Nucleolin (^{19}F)
Nucleolin (^{19}F)
b
Plane #1
Plane #2
Plane #3
Plane #4
Plane #5
Nucleolin (^{19}F)
e⁻
e⁻
Nucleolin (^{19}F)
Plane #6
Plane #7
Plane #8
Plane #9
Plane #10
Nucleolin (^{19}F)
e⁻
e⁻
Nucleolin (^{19}F)

c

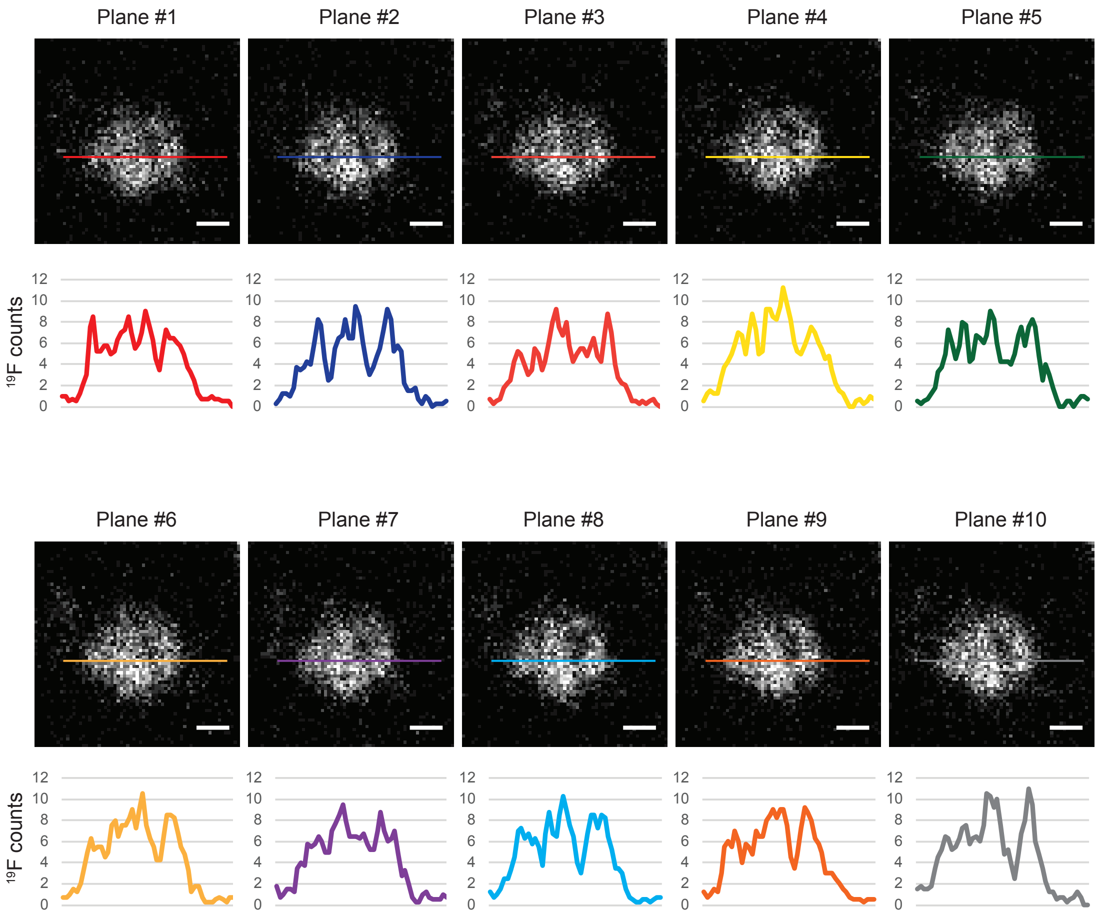

Supplementary Figure 17. Sequential planes are similar in ion counts.

(**A**) Representative HD-MIBI image of a HeLa cell stained with anti-nucleolin-^{19}F/FITC. Scale bars, 4 µm; and 1 µm for enlarged images. n = 3.

(**B**) HD-MIBI images of the 10 individual planes summed in panel A in consecutive order. The ^{19}F signal is similar in all individual images. Scale bars, 4 µm.

(**C**) Line scans on the nucleolus enlarged in panel A for each individual plane. Line scans were used to quantify ion counts per pixel in individual planes. Scale bars, 1 µm.

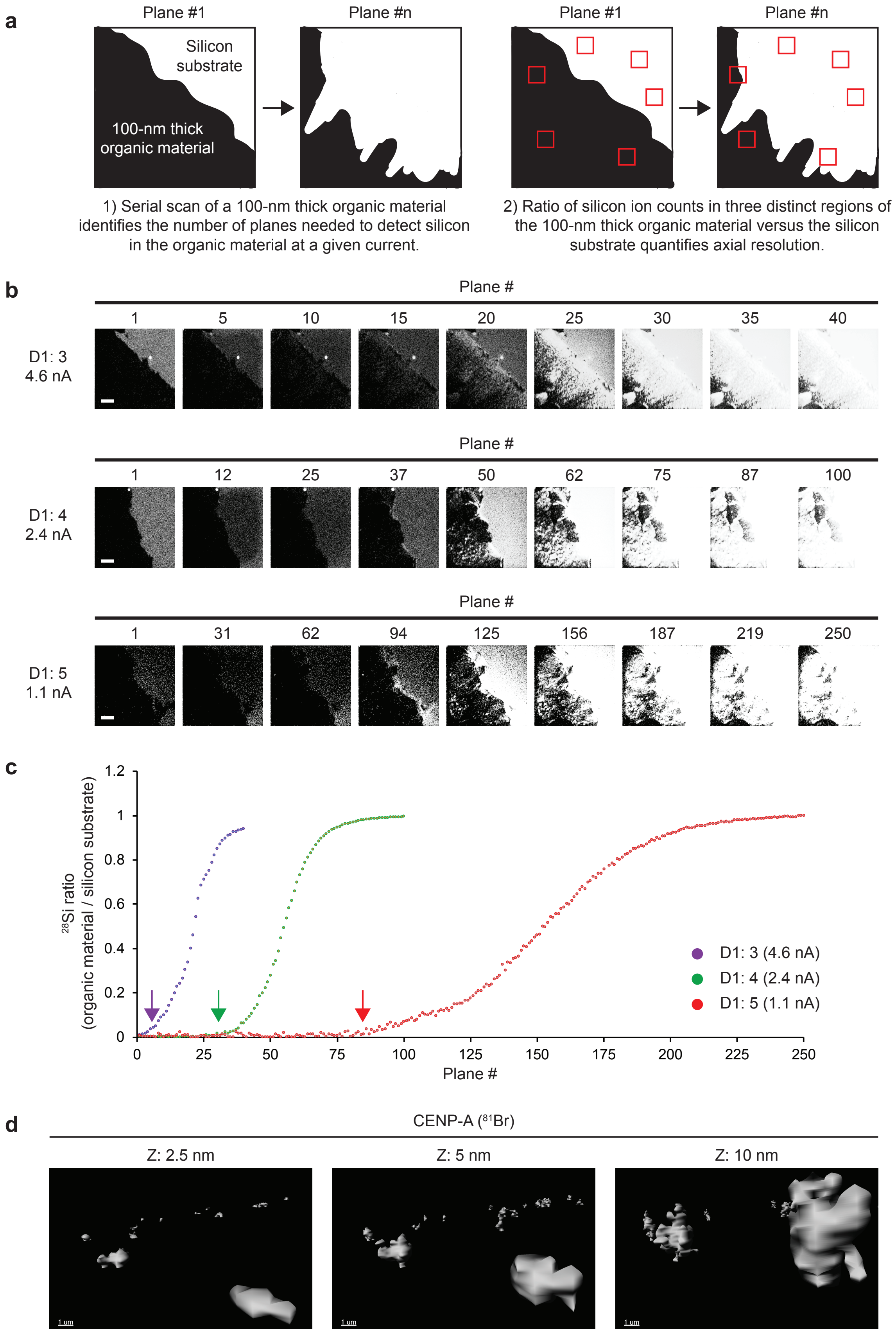
a
Plane #1
Plane #n
Silicon substrate
100-nm thick organic material
1) Serial scan of a 100-nm thick organic material identifies the number of planes needed to detect silicon in the organic material at a given current.
2) Ratio of silicon ion counts in three distinct regions of the 100-nm thick organic material versus the silicon substrate quantifies axial resolution.
b
Plane #
1 5 10 15 20 25 30 35 40
D1: 3
4.6 nA
1 12 25 37 50 62 75 87 100
D1: 4
2.4 nA
1 31 62 94 125 156 187 219 250
D1: 5
1.1 nA
c
28Si ratio
(organic material / silicon substrate)
Plane #
D1: 3 (4.6 nA)
D1: 4 (2.4 nA)
D1: 5 (1.1 nA)
d
CENP-A (81Br)
Z: 2.5 nm
Z: 5 nm
Z: 10 nm

Supplementary Figure 18. Depth profile analysis on 100 nm thick organic material and 3D surface reconstruction of CENP-A signal at 5 nm Z-depth shows the high axial resolution of HD-MIBI.

(**A**) Schematic representation of the workflow of a depth profile analysis on 100 nm thick organic material.

(**B**) Representative images of silicon (^{28}Si) at the edge of a 100 nm thick organic material in the indicated planes. Data acquisition was performed at different currents in independent FOVs until the ^{28}Si intensity in the organic material region was visually indistinguishable from the ^{28}Si intensity in the substrate. Scale bars, 4 µm.

(**C**) Plotting of the ^{28}Si ratio between the organic material regions versus the silicon substrate regions at different planes (six regions per plane as described in A). The arrows show that using D1:3 at 4.6 nA, D1:4 at 2.4 nA and D1:5 at 1.1 nA, about 10 (purple arrow), 30 (green arrow) and 80 (red arrow) planes are required to detect silicon in a 100-nm thick sample, which translates to axial resolutions of about 10, 3.3 and 1.25 nm, respectively.

(**D**) 3D surface reconstruction of CENP-A signal at different Z-depth. HeLa cells were stained with anti-CENP-A-^{81}Br/Cy3, and 40 individual planes were acquired to obtain HD-MIBI images of centromeres from its appearance to its disappearance. 3D surface reconstruction of images of CENP-A at 5 nm reveal centromeres with spherical shape. n = 2.

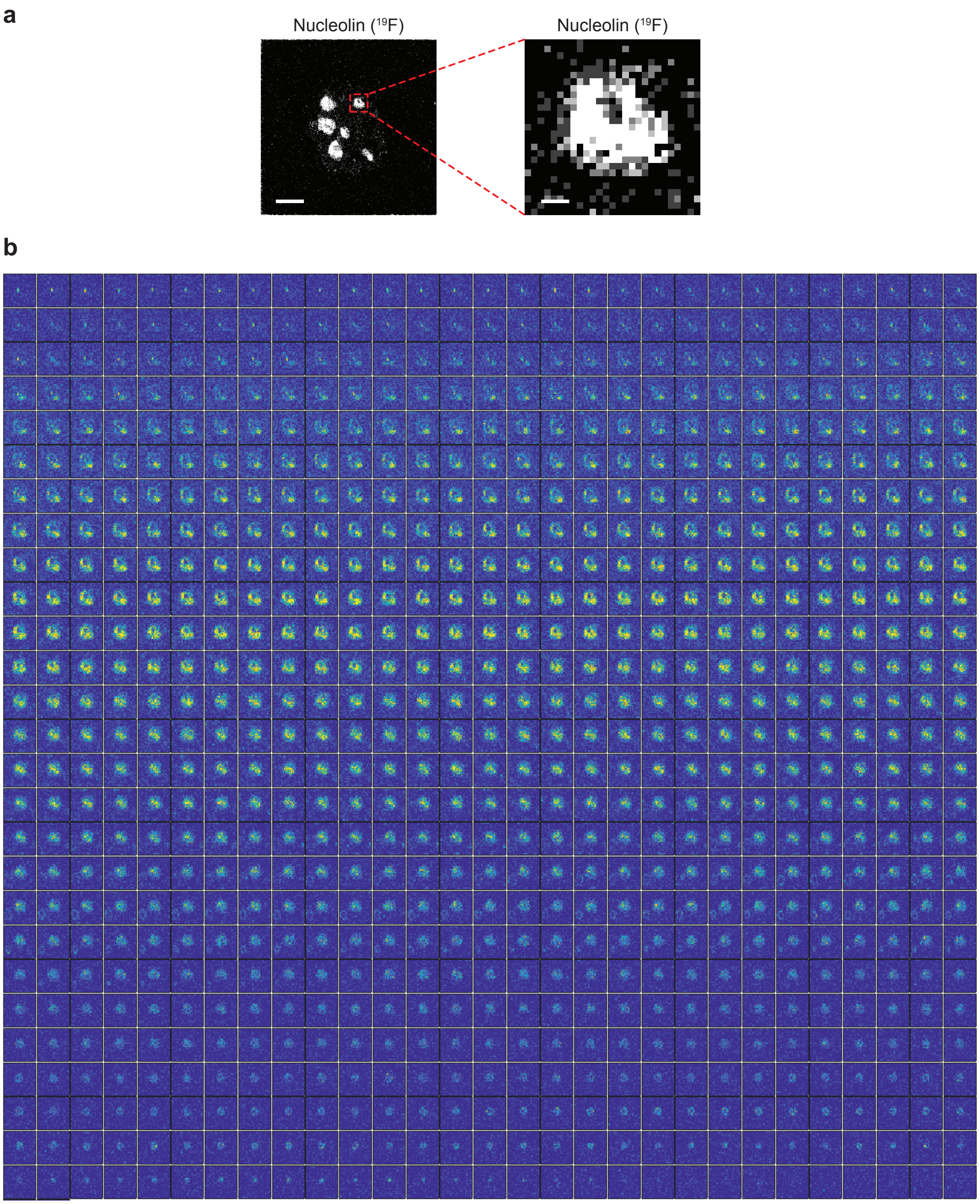

Supplementary Figure 19. Individual planes of a whole nucleolus.

(**A**) (Left) Representative HD-MIBI image of a HeLa cell stained with anti-nucleolin-^{19}F/FITC. Scale bar, 4 μm. (Right) Enlarged image of the nucleolus shown in Fig. 3A-B. Scale bar, 400 mm. n = 3.

(**B**) Individual planes of a whole nucleolus, in order from left to right and from top to bottom; these 783 images were used for the volumetric reconstruction of the nucleolus shown in Fig. 3B. Images have the same scale for intensity, and sequential planes have similar ^{19}F ion counts.

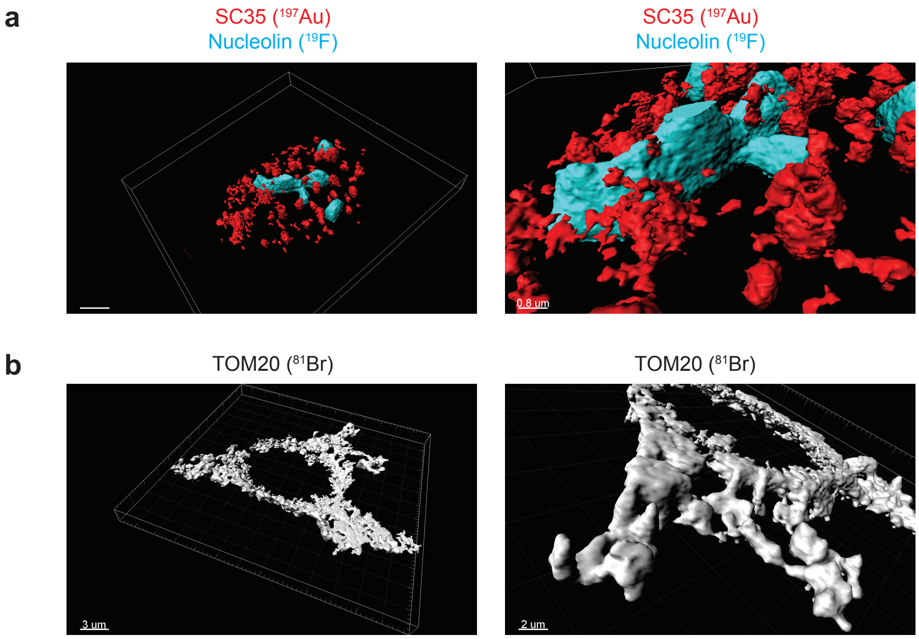

Supplementary Figure 20. 3D reconstruction of the nucleolin HD-MIBI data is different from other subcellular structures.

(**A**) Representative 3D surface reconstruction of nucleolus (cyan) and nuclear speckles (red). HeLa cells were stained with anti-nucleolin-^{19}F/FITC and anti-SC35-biotin (recognized by streptavidin-^{197}Au/FITC). The image consists of the 3D reconstruction of a stack of 204 consecutive planes. n = 3.

(**B**) Representative 3D surface reconstruction of mitochondria. HeLa cells were stained with anti-TOM20-^{81}Br/Cy3. The image consists of the 3D reconstruction of a stack of 631 consecutive planes. n = 1.

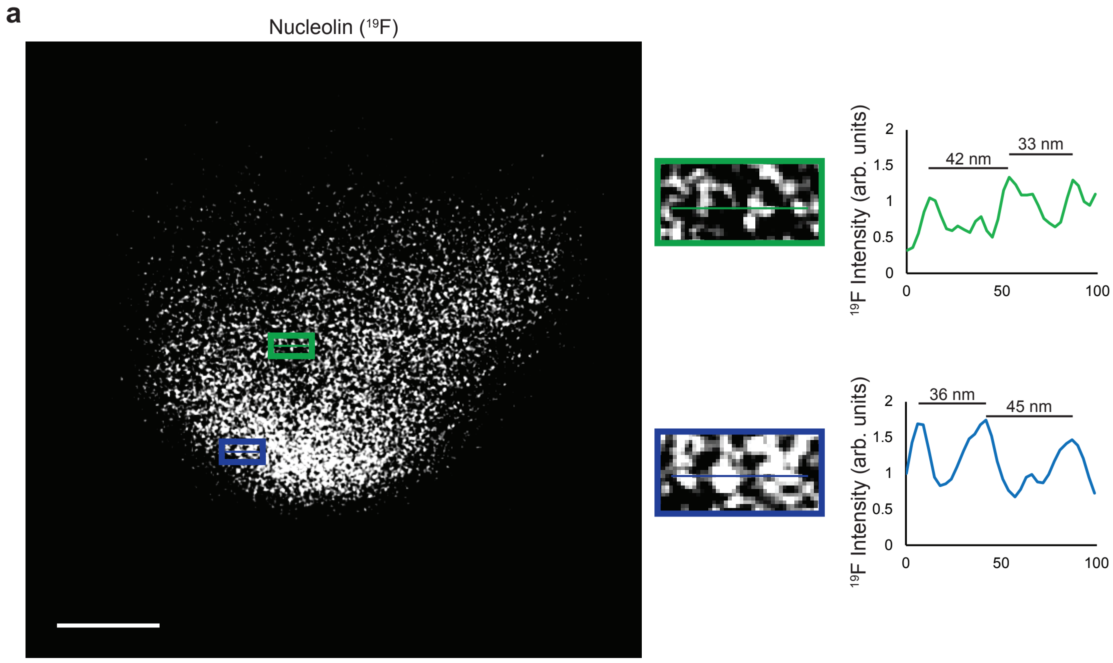

Supplementary Figure 21. Iterative HD-MIBI is able to resolve MoC-Ab signals at the nanoscale.

(Left) Representative enlarged image of a nucleolus of a HeLa cell stained with anti-nucleolin-^{19}F/FITC. The same nucleolus is shown in Fig. 3F. Scale bar, 300 nm. (Middle) Higher magnification images of the area boxed in the image on the left. (Right) Line scans of the lines in the middle images demonstrate that HD-MIBI can resolve signals spaced a few tens of nm. n = 2.

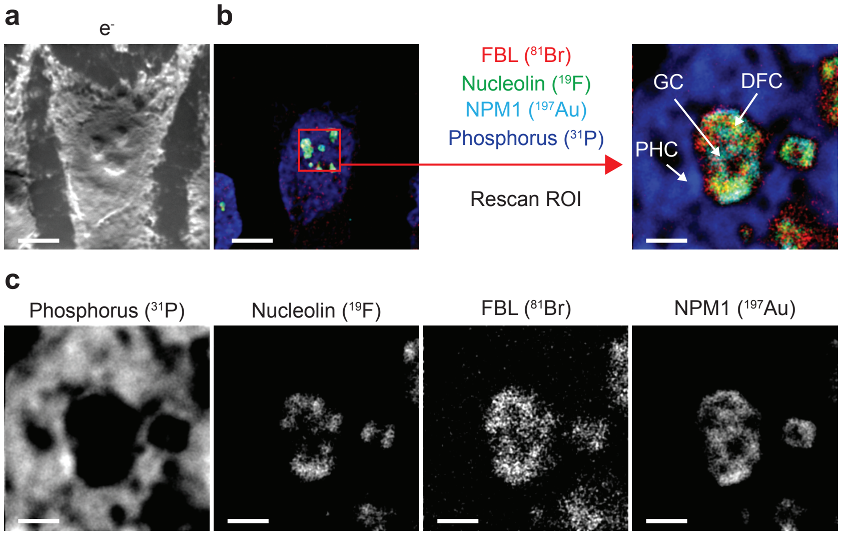

Supplementary Figure 22. Iterative super-resolution imaging reveals nucleolar substructures.

(**A**) Representative secondary electron image of a HeLa cell. Scale bar, 5 µm.

(**B**) (Left) High-resolution scan of the HeLa cell used to find a ROI. The HeLa cell shown in panel A was stained with anti-nucleolin-^{19}F/FITC, anti-FIB-1-^{81}Br/Cy3, and anti-NPM1-biotin (recognized by streptavidin-^{197}Au/FITC). Scale bar, 5 µm. (Right) Iterative super-resolution imaging for the visualization of the nucleolar structure. NPM1, a marker of the granular component (GC), is distributed in regions distinct from those stained with FIB-1, a marker of the dense fibrillary component (DFC). Regions with high phosphorus surrounding the nucleolus indicate perinucleolar heterochromatin (PHC). Scale bar, 1 µm.

(**C**) Individual images of the super-resolution image shown in panel B. Scale bars, 1 µm.

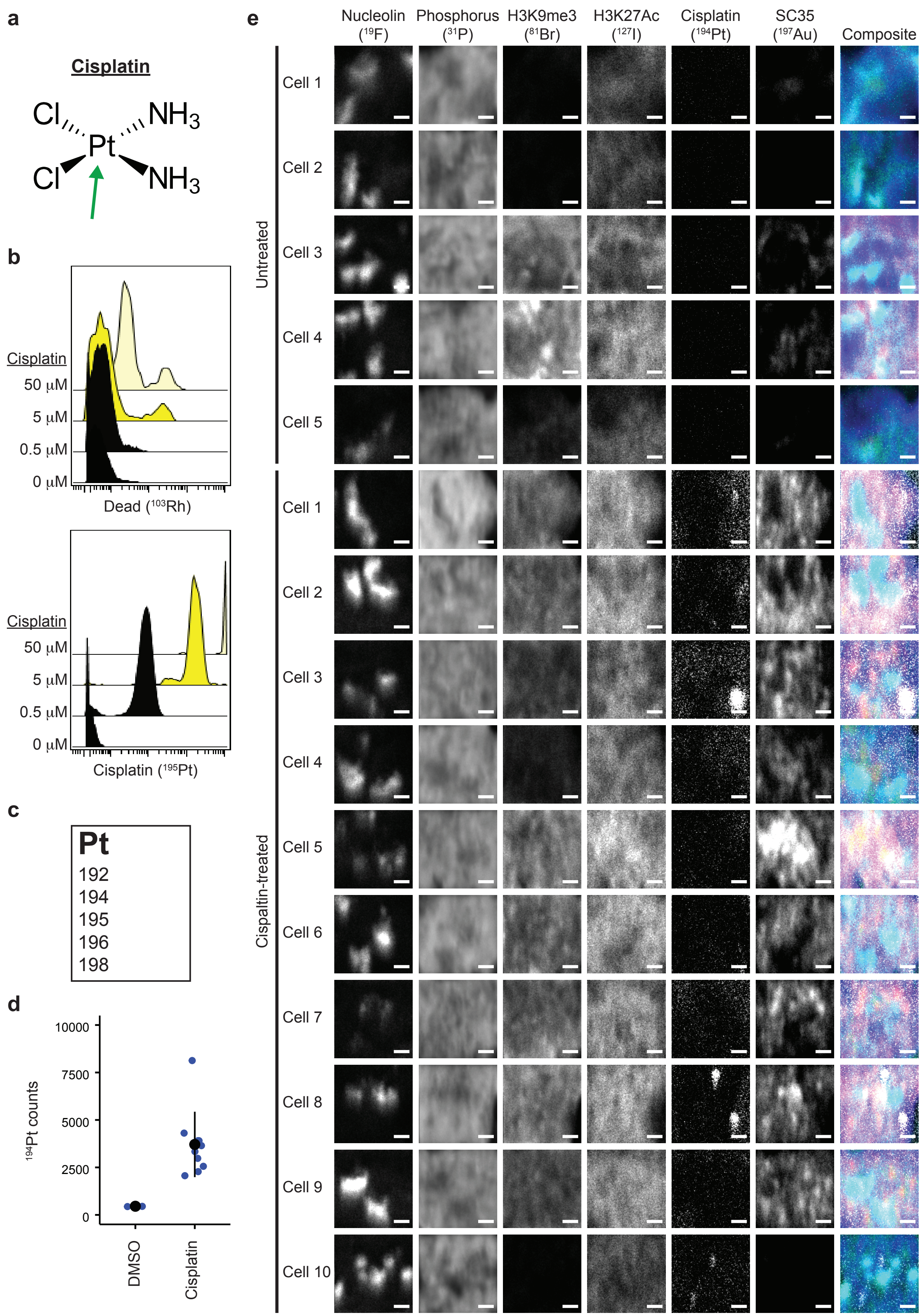

a
Cisplatin
Cl
Cl
Pt
NH3
NH3
b
Cisplatin
50 µM
5 µM
0.5 µM
0 µM
Dead (103Rh)
Cisplatin
50 µM
5 µM
0.5 µM
0 µM
Cisplatin (195Pt)
c
Pt
192
194
195
196
198
d
194Pt counts
10000
7500
5000
2500
0
DMSO
Cisplatin
e
Nucleolin (19F)
Phosphorus (31P)
H3K9me3 (81Br)
H3K27Ac (127I)
Cisplatin (194Pt)
SC35 (197Au)
Composite
Untreated
Cell 1
Cell 2
Cell 3
Cell 4
Cell 5
Cispaltin-treated
Cell 1
Cell 2
Cell 3
Cell 4
Cell 5
Cell 6
Cell 7
Cell 8
Cell 9
Cell 10

Supplementary Figure 23. Validation of cisplatin for HD-MIBI.

(**A**) Chemical structure of cisplatin.

(**B**) CyTOF analysis of cisplatin distribution in TYK-nu ovarian cancer cells. TYK-nu cells were treated with different concentrations of cisplatin for 24 hours, washed, and treated with 1 μM Rh-intercalator for 15 minutes (to discriminate dead from live cells) and analyzed by CyTOF. Maximal drug uptake with minimal cell death at 24 hours was observed with 5 μM.

(**C**) The five naturally occurring stable isotopes of cisplatin.

(**D**) Mean of ion count per pixel of all pixels in HD-MIBI images from TYK-nu cells treated with cisplatin. TYK-nu cells were treated with DMSO (n=5) or 15 μM cisplatin (n=10) for 72 hours and analyzed by HD-MIBI. Minimal signal was observed in DMSO-treated cells in the ^{194}Pt channel. Data are presented as mean values (black dots) +/- SD. Data points (blue dots) are overlaid.

(**E**) Assessment of ^{194}Pt channel crosstalk. HD-MIBI images of TYK-nu cells treated with DMSO or 15 μM cisplatin for 72 hours and stained with anti-nucleolin-^{19}F, anti-H3K9me3-^{81}Br, anti-H3K27Ac-^{127}I, and anti-SC35-biotin (recognized by streptavidin-^{197}Au). Each column represents the ion map for the indicated isotope. Images consist of the sums of 20 consecutive planes. Scale bars, 1 μm. n = 5 cells for DMSO, and n = 10 cells for cisplatin in 1 experiment.

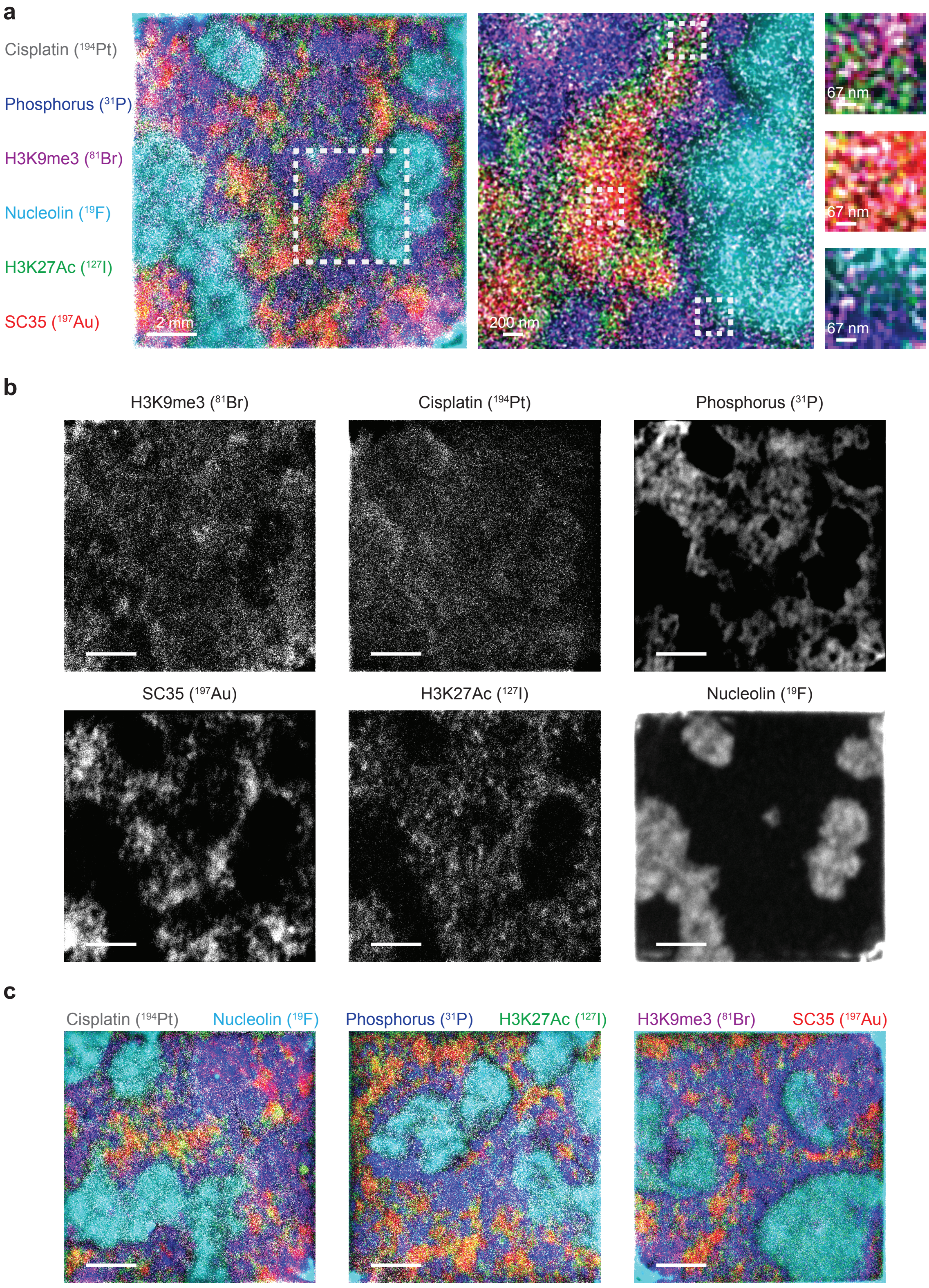

a
Cisplatin (194Pt)
Phosphorus (31P)
H3K9me3 (81Br)
Nucleolin (19F)
H3K27Ac (127I)
SC35 (197Au)
2 mm
200 nm
67 nm
67 nm
67 nm
b
H3K9me3 (81Br)
Cisplatin (194Pt)
Phosphorus (31P)
SC35 (197Au)
H3K27Ac (127I)
Nucleolin (19F)
c
Cisplatin (194Pt)
Nucleolin (19F)
Phosphorus (31P)
H3K27Ac (127I)
H3K9me3 (81Br)
SC35 (197Au)

Supplementary Figure 24. Six-channel HD-MIBI images on TYK-nu cells treated with cisplatin.

(**A**-**C**) Representative HD-MIBI images of TYK-nu cells treated with 5 µM cisplatin for 24 hours and stained with anti-nucleolin-^{19}F/FITC, anti-H3K9me3-^{81}Br/Cy3, anti-H3K27Ac-^{127}I/Cy5, and anti-SC35-biotin (recognized by streptavidin-^{197}Au). Images of nucleolin (^{19}F; cyan), DNA (^{31}P; blue), H3K9me3 (^{81}Br; magenta), H3K27Ac (^{127}I; green), cisplatin (^{194}Pt; grey), and SC35 (^{197}Au; red) were simultaneously acquired. (**A**) A composite image of a TYK-nu cell nucleus. Scale bars, 2 µm (right image), 200 nm (middle image) and 67 nm (left images). (**B**) Individual images of the cell shown in panel A. Scale bars, 2 µm. (**C**) Composite images from three additional TYK-nu cell nuclei. Scale bars, 2 µm. n = 5 cells examined in 1 experiment.

a

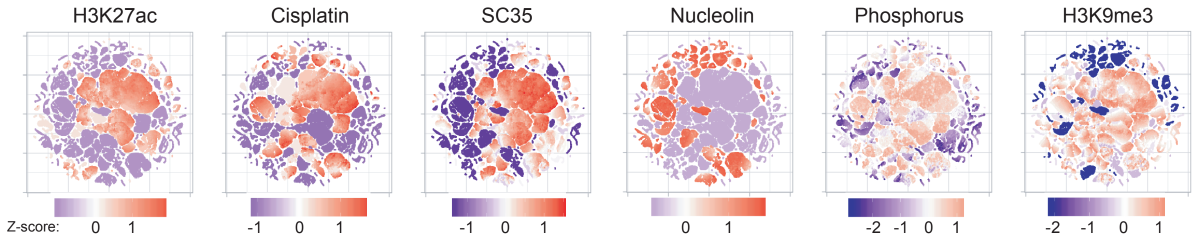

b

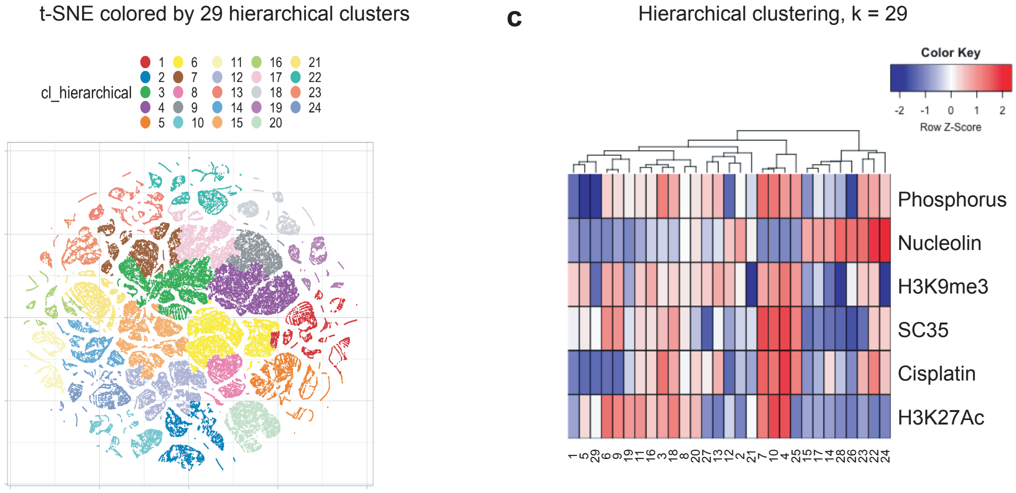

d

Manual annotation and merging of clusters

k = 29			
Annotation	Clusters	New cluster Num	Events per cluster
Heterochromatin 1	1, 5	1	8597
Heterochromatin 2	13, 27	2	8753
Euchromatin 1	11	3	2876
Euchromatin 2	16, 19	4	9378
Euchromatin + Cisplatin	3, 18	5	10017
Active SC35 + Cisplatin	4, 7, 10	6	10497
Inactive SC35 + Ac	25	7	24467
SC35	6, 9	8	13418
Nucleolin	2, 12, 14, 15, 17, 23, 24, 26, 28	9	25003
Nucleolin + Cisplatin	22	10	3495
Others	20, 21, 29	11	5499

e

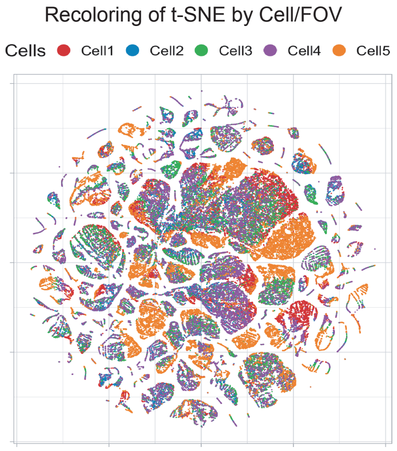

Supplementary Figure 25. Identification of nuclear neighborhoods by the iterative HD-MIBI analysis framework.

(**A**) t-SNE was performed on 100000 voxels of dimensions (x, y, z) = (10, 10, 5) pixels (20000 voxels from each cell were randomly sampled across 5 different cells). Each point represents a voxel, consisting of the expression of six different parameters (phosphorus, nucleolin, H3K9me3,

SC35, cisplatin, and H3K27Ac). Voxels grouped into distinct regions based on similar combinatory expression of each marker. The t-SNE map was recolored from left to right to represent the scaled intensity (Z-score) of each indicated marker.

(**B**) Unsupervised hierarchical clustering was performed on the voxels to separate them into 29 distinct groups; the t-SNE map from panel A is shown recolored based on these groups.

(**C**) A heatmap representation of the 29 distinct clusters identified based on the average expression per cluster of SC35, H3K9me3, phosphorus, nucleolin, H3K27Ac, and cisplatin. The scale intensity of each marker is denoted by the color bar on the top right (Z-score, normalized to each row).

(**D**) Clusters were manually merged by tree distance, proximity on the t-SNE map and similar expression profiles. This resulted in a reduction of 29 clusters into 11 clusters, which were then annotated based on their final average expression profile.

(**E**) t-SNE map from panel A colored by the cell of origin (from 5 different cells).

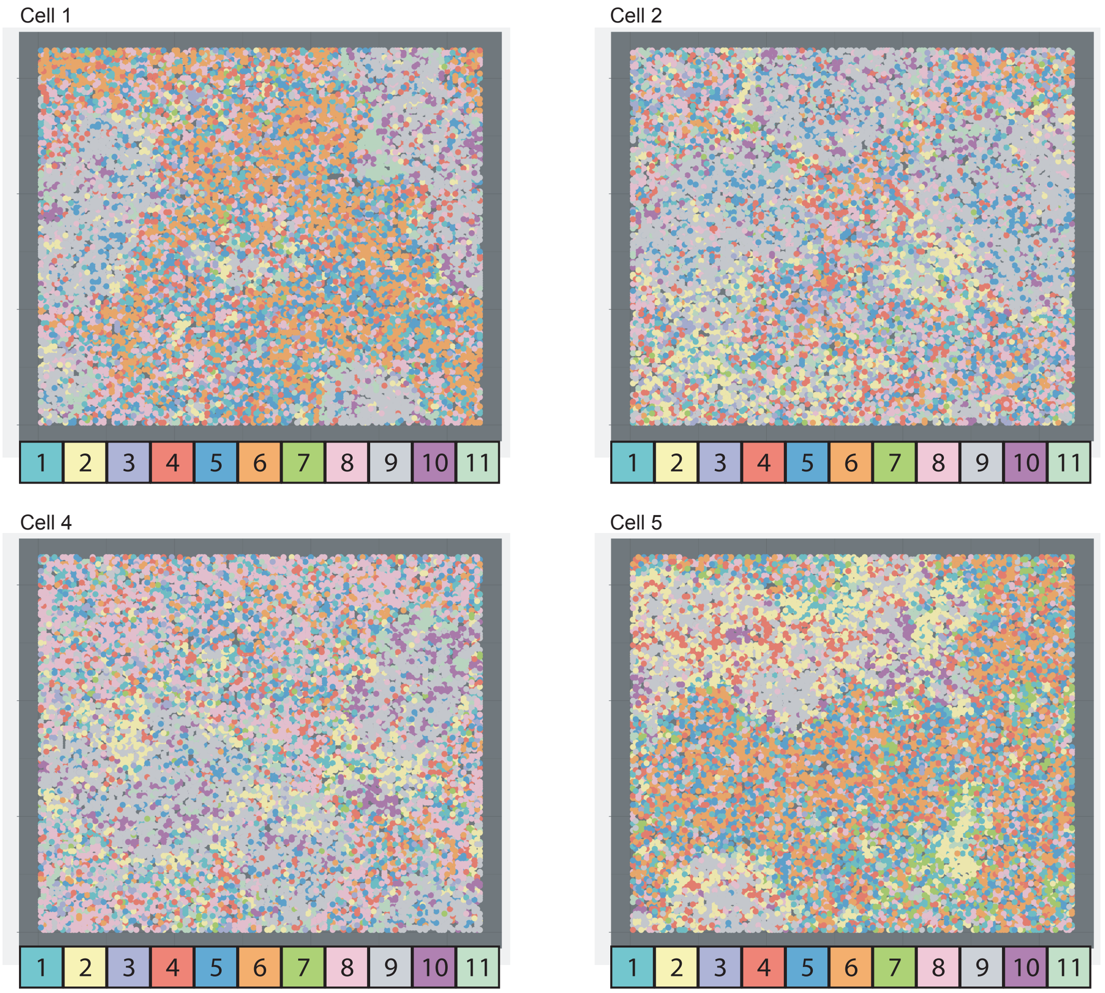

Supplementary Figure 26. The spatial distribution of nuclear neighborhoods across cells reveals order and diversity in nuclear organization.

Nuclear neighborhoods were recolored by each cluster in each of five cells to allow a visual understanding of nuclear neighborhood interactions and conserved and divergent features. Cells 1, 2, 4, and 5 are shown here. Cell 3 is shown in Fig. 5F.

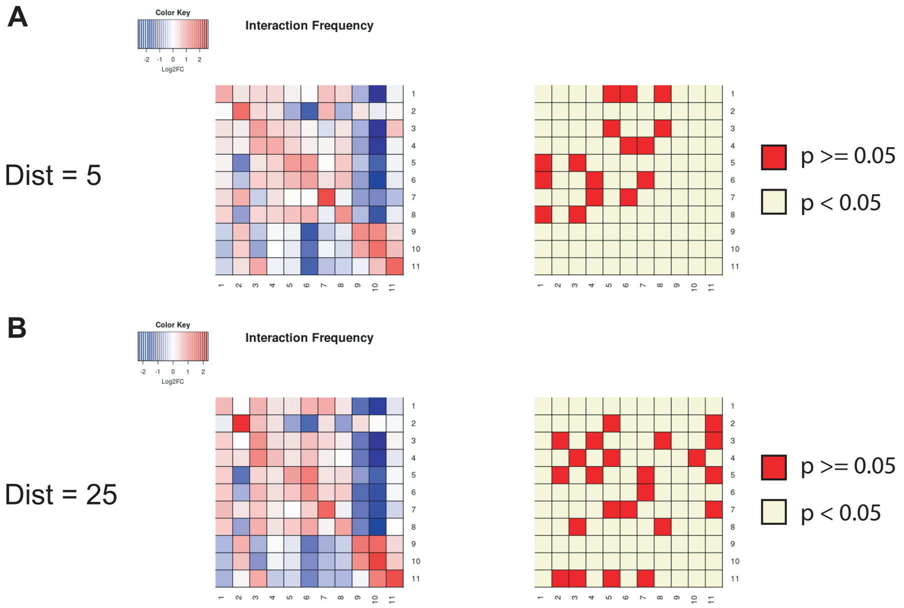

Supplementary Figure 27. Pairwise neighborhood interaction frequency at different cutoffs. Pairwise voxels were defined as neighbors using a cutoff of *n* pixels in 3D Euclidean space (see Materials and Methods for details). We tested two distances here, using (**A**) 5 pixels and (**B**) 25 pixels as the cutoff. The neighborhood interaction frequency map was calculated using the log2 enrichment of the real over expected number of pairwise interaction frequencies (left). The adjusted p-values are plotted as significant ($p < 0.05$, cream) or not significant ($p >= 0.05$, red) (right).

a

b

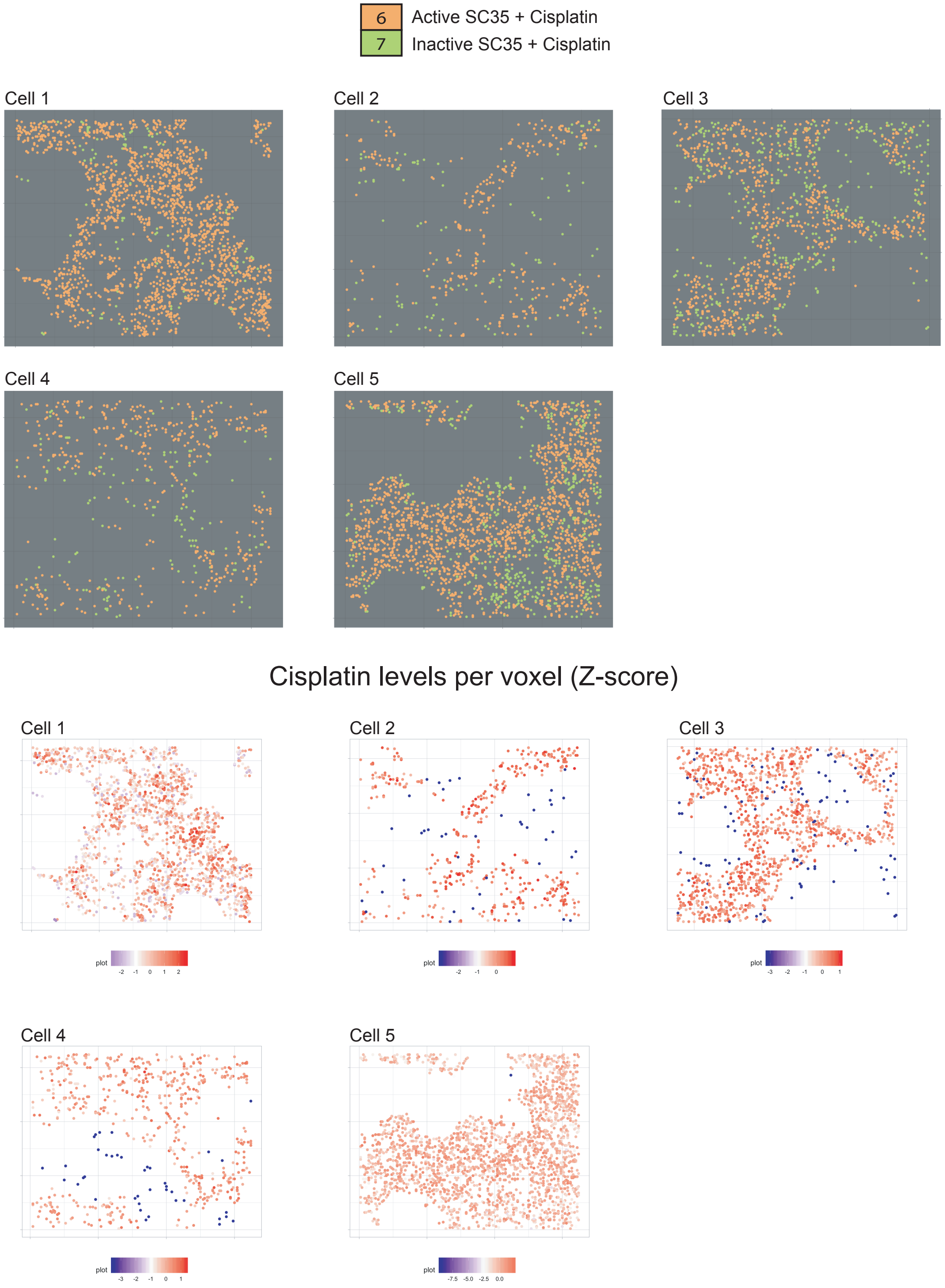
6 Active SC35 + Cisplatin
7 Inactive SC35 + Cisplatin
Cell 1
Cell 2
Cell 3
Cell 4
Cell 5
Cisplatin levels per voxel (Z-score)
Cell 1
Cell 2
Cell 3
plot -2 -1 0 1 2
plot -2 -1 0
plot -3 -2 -1 0 1
Cell 4
Cell 5
plot -3 -2 -1 0 1
plot -7.5 -5.0 -2.5 0.0

Supplementary Figure 28. Identification of a cisplatin gradient within SC35 nuclear speckles.

(**A**) Neighborhoods 6 (Active SC35 and Cisplatin) and 7 (Inactive SC35 and Cisplatin) were plotted for all 5 cells, to represent the spatial distribution of these two neighborhoods relative to each other.

(**B**) Scaled cisplatin levels (Z-score) were plotted on neighborhoods 6 and 7. An increasing gradient of cisplatin concentration is observed across the inactive-active SC35 transition boundary.

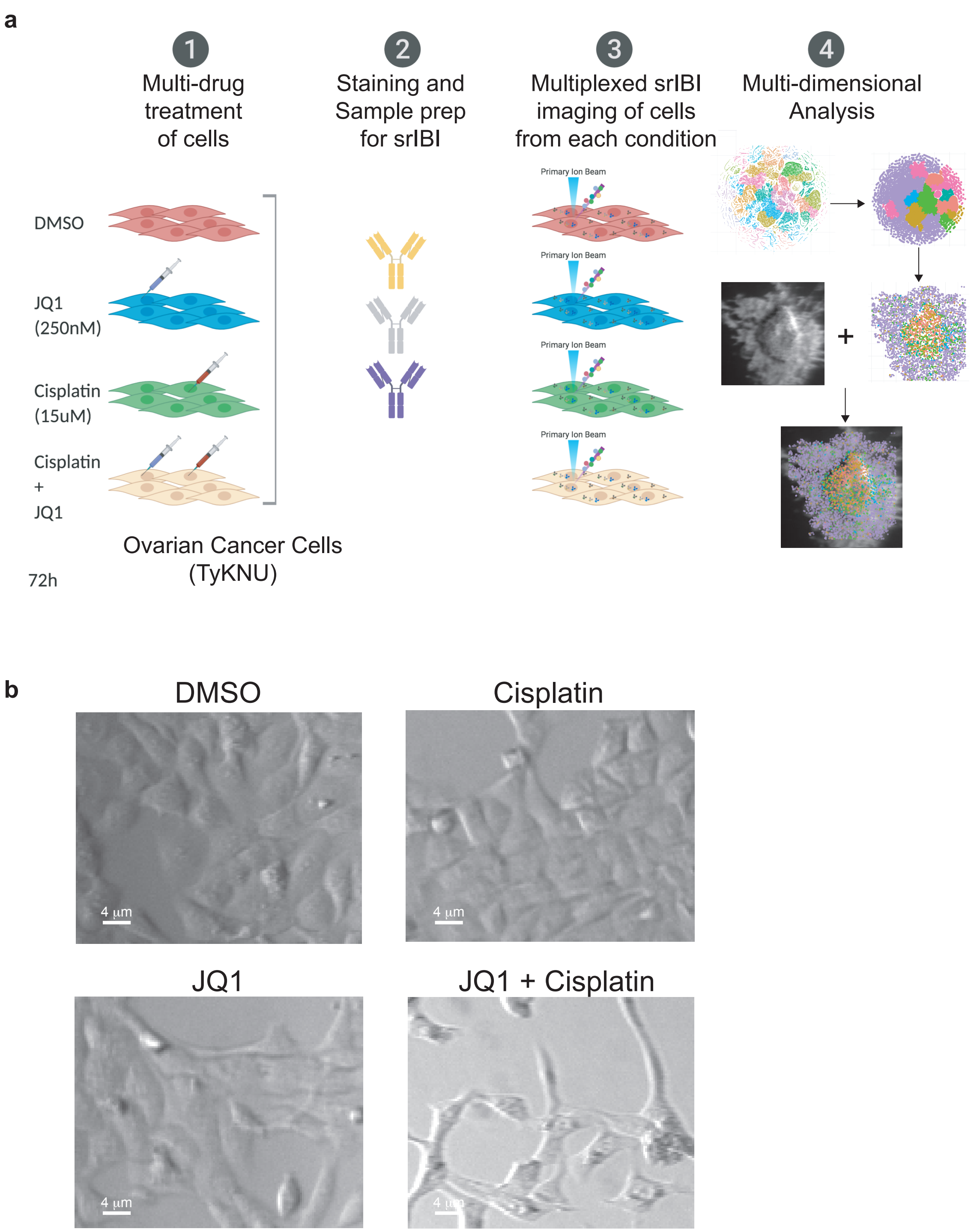

Supplementary Figure 29. Experimental workflow for studying multi-drug resistance in Ovarian Cancer cells.

(**A**) An overview of the experimental workflow. (1) TYK-nu ovarian cancer cells were treated with cisplatin and JQ1 individually or in combination, (2) followed by subsequent sample preparation

for HD-MIBI imaging with anti-nucleolin-^{19}F/FITC, anti-H3K9me3-^{81}Br/Cy3, anti-H3K27Ac-^{127}I/Cy5, and anti-SC35-biotin (detected with streptavidin-^{197}Au/FITC). (3) Multiple cells from each condition (DMSO: n = 8, JQ1: n = 6, Cisplatin: n = 10, JQ1 + Cisplatin: n = 9) were imaged, followed by a multi-dimensional analysis.

(**B**) Representative bright field images of cells 72 hours post-treatment. Cells were seeded at the same density before drug treatment. Scale bars, 4 μm. n = 3.

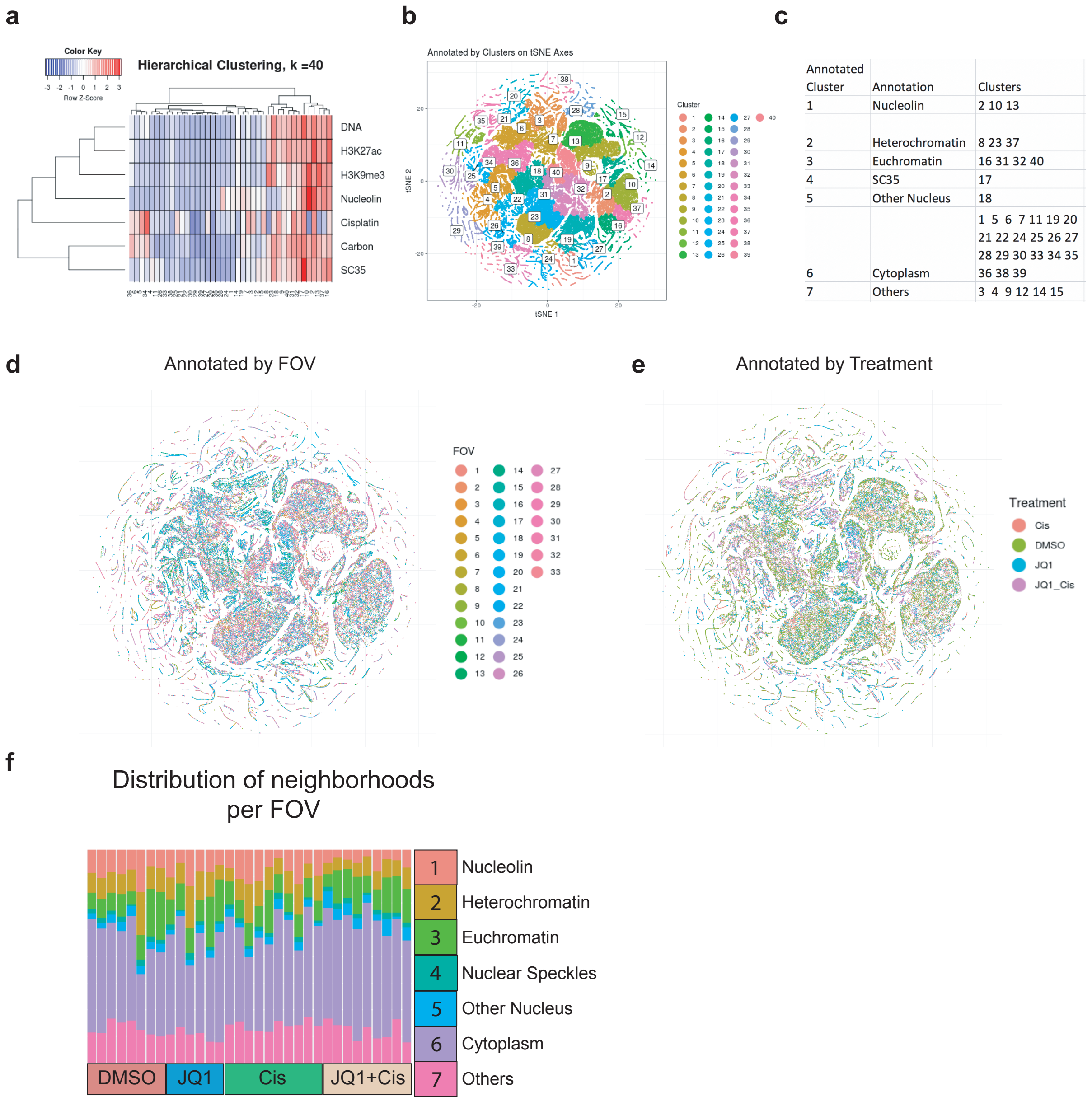

Supplementary Figure 30. Dimensional reduction and hierarchical clustering of cellular neighborhoods.

(**A**) Hierarchical clustering was performed on Euclidean distance between the sampled voxels projected onto a two-dimensional t-SNE space from Figure 6C. The dendrogram was cut into 40 clusters, and the mean expression of each marker is plotted as Z-score.

(**B**) Clusters from panel A were colored on the t-SNE plot.

(**C**) Clustered were manually combined to identify cellular neighborhoods in Figure 6C, based on their mean expression in panel A as well as spatial distributions.

(**D**) Each voxel plotted in t-SNE space was colored by the FOV.

(**E**) Each voxel plotted in t-SNE space was colored by treatment.

(**F**) Neighborhood distributions for each FOV is plotted, with its treatment conditions indicated on the bottom.

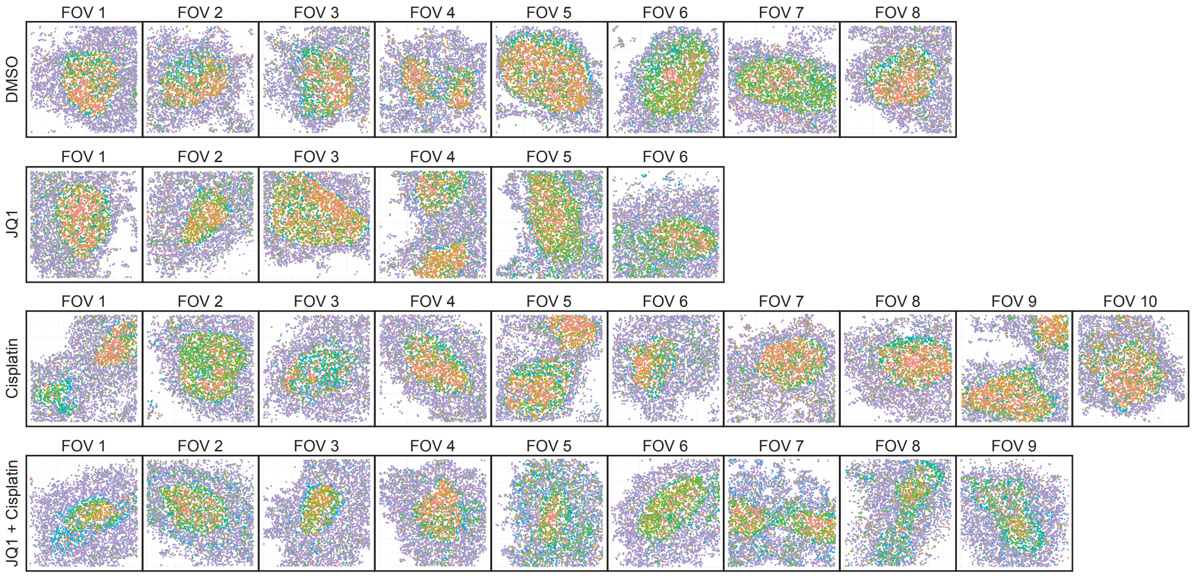

Supplementary Figure 31. Recreation of each FOV through Cellular neighborhood plots. Cellular neighborhoods were plotted for each FOV as per the annotation in Figure 6C and Figure S29.

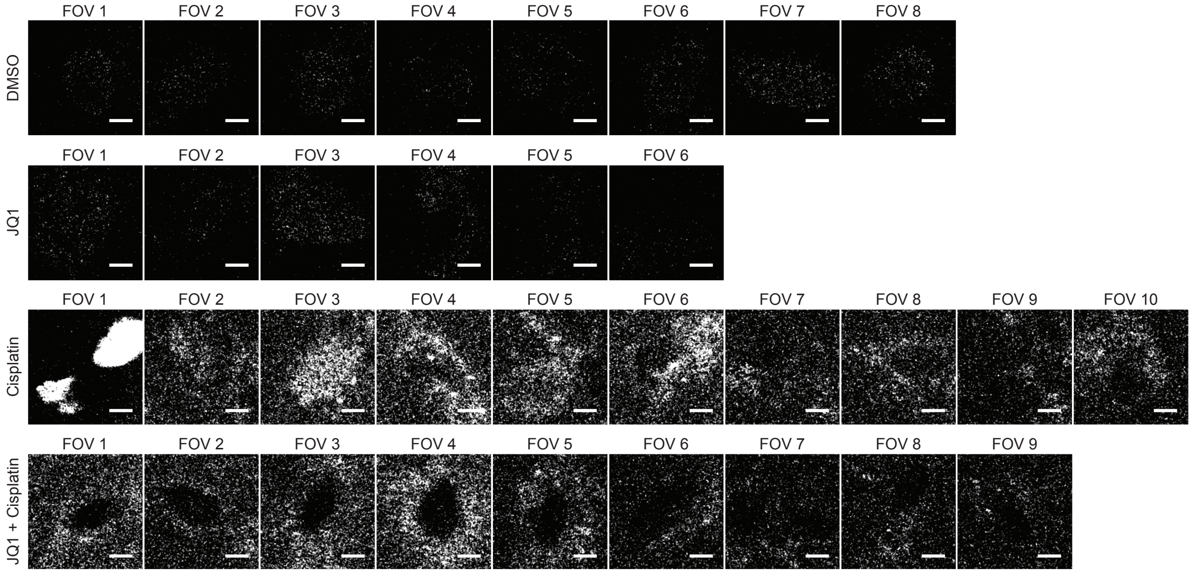

Supplementary Figure 32. Distribution of cisplatin for each FOV.

Cisplatin counts were plotted for each FOV, for each treatment. All 33 images were normalized on the same scale, before a gaussian blur with radius = 1 before plotting. Scale bars, 3 µm. n = 8 cells for DMSO, n = 6 cells for JQ1, n = 10 cells for cisplatin, and n = 9 cells for JQ1 + cisplatin examined in 1 experiment.

a

Radius = 2

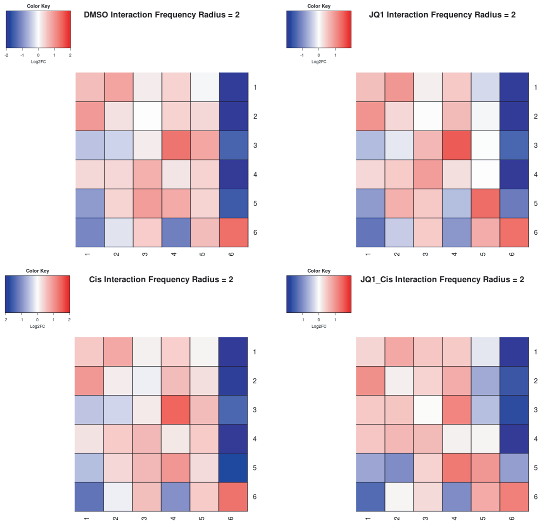

b

Radius = 5

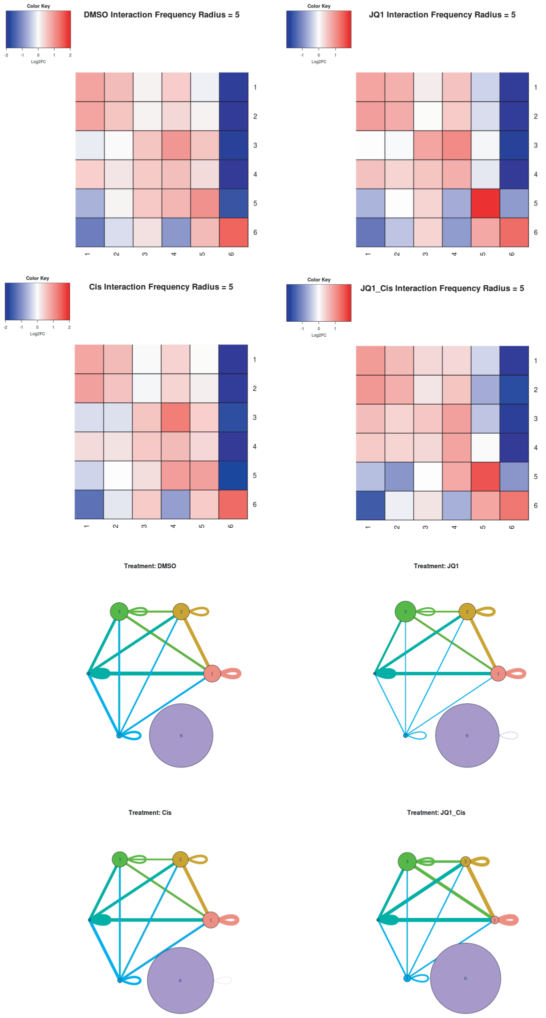

Supplementary Figure 33. Neighborhood interaction plots at various radii.

(**A**) Neighborhood enrichment (log2 observed over expected) is represented for radius = 2 voxels, and (**B**) radius = 5 voxels.

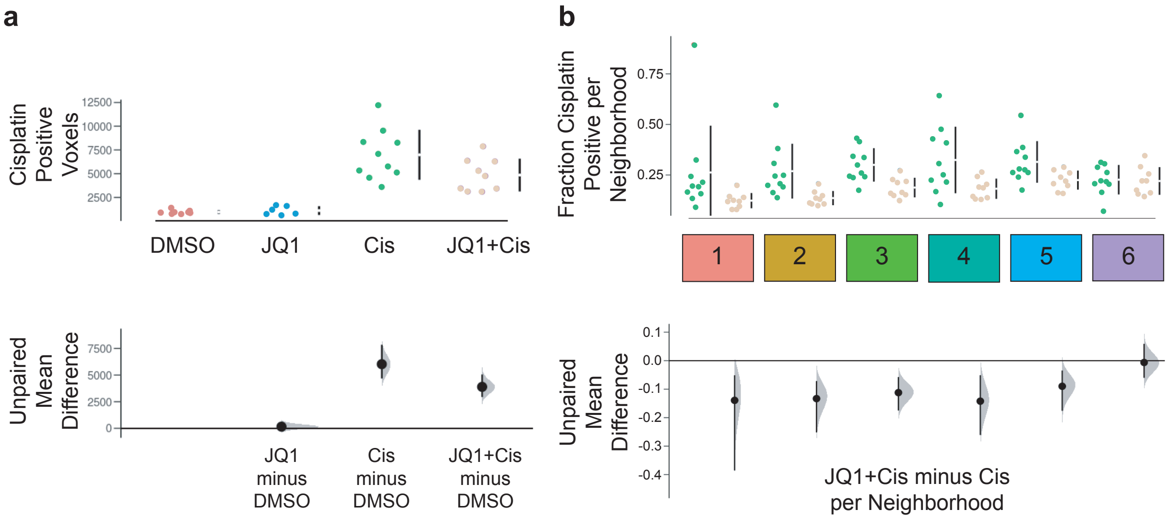

Supplementary Figure 34. Neighborhood composition statistics.

(**A**) All voxels from each FOV across all 4 conditions are plotted in a Cumming plot for whether they were positive for cisplatin, showing the mean differences and bootstrapped 95% confidence intervals of each condition compared to control (DMSO).

(**B**) The fraction of sampled voxels from each of the 6 annotated neighborhoods identified in Fig. 5A that were positive for cisplatin counts are plotted in a Cumming plot, showing the mean differences and 95% confidence intervals of JQ1 + cisplatin compared to cisplatin alone. Each dot is a cell.

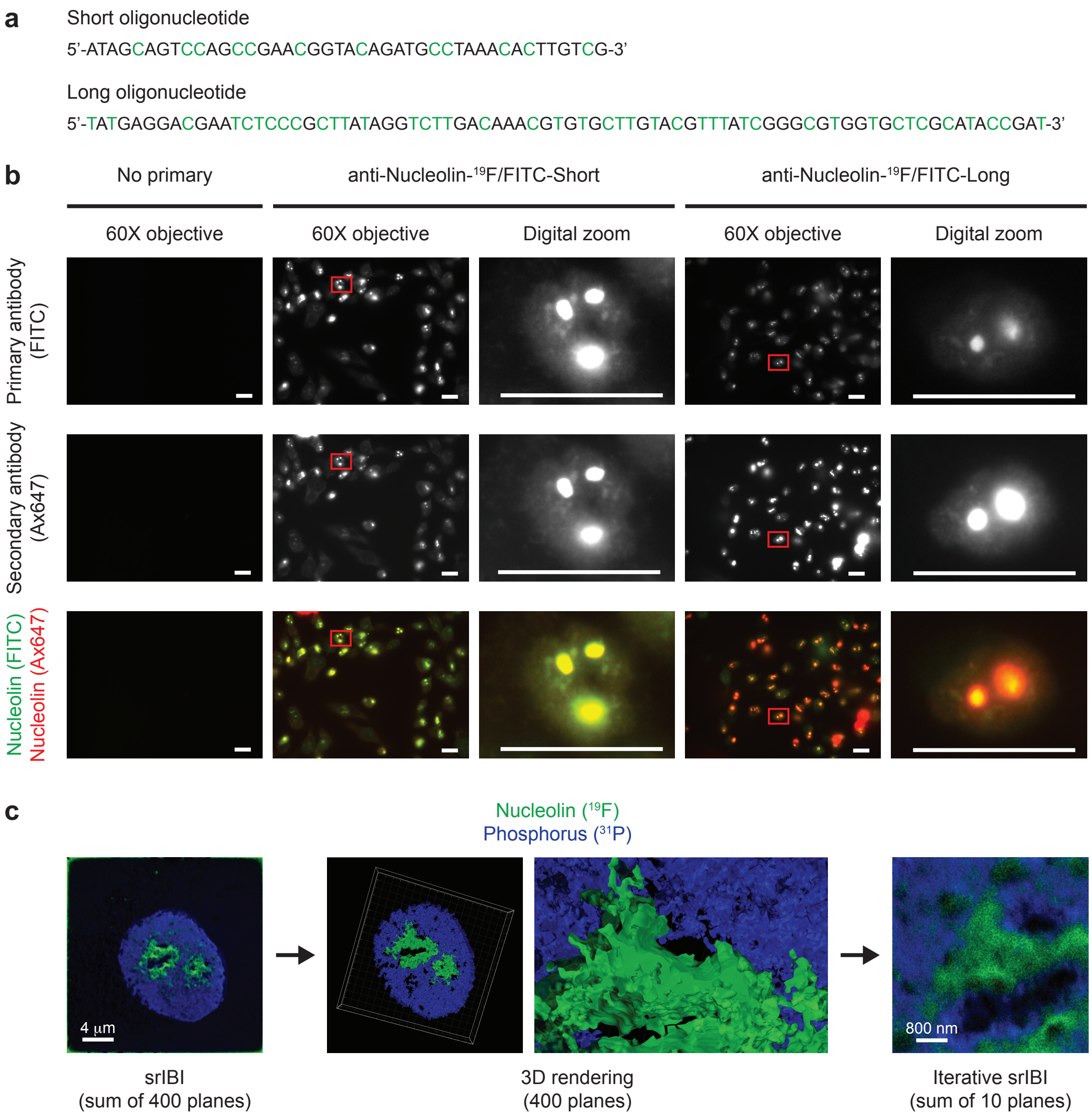

Supplementary Figure 35. A ~two-fold longer oligonucleotide is suitable for high-resolution imaging using HD-MIBI.

(**A**) Sequence for the short and long oligonucleotides. Each antibody has about 4 to 6 binding sites. In this work, we have conjugated antibodies to oligonucleotides consisting of 43 nucleotides (short oligonucleotide). Each short oligonucleotide contains 12 labeled nucleotides (green nucleotides in the short oligonucleotide) for a total of 48 to 72 isotopic labels per antibody. The new

oligonucleotide consists of 80 nucleotides (long oligonucleotide). Each long oligonucleotide contains 42 labeled nucleotides (green nucleotides in the long oligonucleotide) for a total of 168 to 252 isotopic labels per antibody.

(**B**) Representative confocal image of HeLa cells stained with anti-nucleolin-^{19}F/FITC-Short or anti-nucleolin-^{19}F/FITC -Long (green) and a secondary anti-mouse-Alexa647 (red). Scale bar, 20 µm. n=1.

(**C**) Representative HD-MIBI, 3D rendering and iterative HD-MIBI images of the same HeLa cell stained with anti-nucleolin-^{19}F/FITC -Long (green).

Supplementary Table 1. Oligonucleotides.

Oligo Name	Sequence (5' → 3')	5'	3'	Z-Modification
Det.F.6-FAM	ATAGZAGTZZAGZZGAAZGGTAZ AGATGZZTAAAZAZTTGTZG	Maleimide	6-FAM	2'-F-Ac-C
Det.Br.Cy3	ATAGZAGTZZAGZZGAAZGGTAZ AGATGZZTAAAZAZTTGTZG	Maleimide	Cy3	5-Br-dC
Det.I.Cy5	ATAGZAGTZZAGZZGAAZGGTAZ AGATGZZTAAAZAZTTGTZG	Maleimide	Cy5	5-I-dC

Supplementary Table 2. Primary antibodies.

Antigen	Catalog number; Provider	Clone	Tag	Staining concentration
dsDNA	ab27156; Abcam	35I9	^{162}Dy	1:50
dsDNA	ab27156; Abcam	35I9	^{127}I	1:50
nucleolin	ADI-KAM-CP100-E; Enzo	4E2	^{19}F / 6-FAM	1:100
H3K27Ac	ab4729; Abcam	pAb	^{19}F / 6-FAM	1:100
CENP-A	ab13939; Abcam	3-19	^{81}Br / Cy3	1:500
H3K27Ac	ab4729; Abcam	pAb	^{127}I / Cy5	1:100
SC35	556363; BD Biosciences	αSC35	Biotin	1:100
H3K9me3	ab8898; Abcam	pAb	^{81}Br / Cy3	1:100
nucleolin	ADI-KAM-CP100-E; Enzo	4E2	^{127}I / Cy5	1:100
NPM1	MABE937; EMD Millipore	28M1	Biotin	1:100
FBL	MABE1154; EMD Millipore	38F3	^{81}Br / Cy3	1:50

Supplementary Table 3. Buffers for intracellular staining.

Buffer	Composition	Catalog number; Provider
Fixation / Permeabilization	1X Fixation/Permeabilization concentrate<br>in Fixation/Permeabilization diluent	00-5123; ThermoFisher<br>00-5223; ThermoFisher
Wash Buffer	1X Permeabilization buffer in water	00-8333; ThermoFisher
Block 1	1X Permeabilization buffer<br>0.5% BSA<br>0.5 M NaCl<br>200 µg/mL Salmon Sperm DNA<br>10 µg/ml Human FcX Block<br>in avidin buffer	00-8333; ThermoFisher<br>A3059; Sigma-Aldrich<br>AM9760G; ThermoFisher<br>AM9680; ThermoFisher<br>564765; BD Biosciences<br>927301; BioLegend
Block 2	1X Permeabilization buffer<br>0.5% BSA<br>0.5 M NaCl<br>200 µg/mL Salmon Sperm DNA<br>10 µg/ml Human FcX Block<br>in biotin buffer	00-8333; ThermoFisher<br>A3059; Sigma-Aldrich<br>AM9760G; ThermoFisher<br>AM9680; ThermoFisher<br>564765; BD Biosciences<br>927301; BioLegend
Reaction	1X Permeabilization buffer<br>0.5% BSA<br>0.5 M NaCl<br>200 µg/mL Salmon Sperm DNA<br>in water	00-8333; ThermoFisher<br>A3059; Sigma-Aldrich<br>AM9760G; ThermoFisher<br>AM9680; ThermoFisher
Post-Fixation	2% Glutaraldehyde in 1X PBS	G7526; Sigma-Aldrich

Supplementary Table 4. Raster size, pixel number, plane number, and total scan time for each cell.

Cell in figure	Raster size (μm)	Pixel number	Plane number	Aperture (D1)	Total scan time (s)
1A	25	512*512	160	5	41966
1C	25	256*256	10	3	678
1D	25	256*256	10	3	678
1E (red dot)	25	512*512	10	2	2644
1E (orange dot)	25	512*512	10	3	2644
1E (yellow dot)	25	512*512	10	4	2644
1E (green dot)	25	512*512	10	5	2644
2A	25	256*256	10	3	678
2B	25	256*256	10	3	678
2C	25	256*256	50	3	3390
2D-H, J	25	512*512	10	5	2644
3A, B	25	256*256	785	3	53223
3C	25	256*256	40	3	2712
3D	25	256*256	400	3	27120
3E (top)	25	256*256	10	3	678
3E (bottom)	10	1024*1024	1	3	1048
3F	3	1024*1024	1	5	1048
3G	10	512*512	200	5	52451
4A	25	256*256	100	3	6780

5A	10	1024*1024	20	5	20960
6A	15	256*256	40	2	2712
S3D (left)	25	256*256	10	3	678
S3D (right)	25	256*256	10	3	678
S4A	25	256*256	100	3	6780
S5A-C (related to 1D)	25	256*256	10	3	678
S6 (related to 1E)	25	512*512	10	2 / 3 / 4 / 5	2644
S7D (left)	25	256*256	10	3	678
S7D (right)	25	256*256	10	3	678
S8D (left)	25	256*256	10	3	678
S8D (right)	25	256*256	10	3	678
S9A	25	256*256	10	3	678
S9B	25	256*256	20	3	1356
S9C	25	256*256	140	3	9492
S10B (top)	60	256*256	50	1	3390
S10B (middle)	30	512*512	49	3	12956
S11C	25	256*256	170	3	11526
S12D (left)	25	256*256	10	3	678
S12D (right)	25	256*256	10	3	678
S13	25	256*256	10	3	678
S14	25	256*256	10	3	678
S15 (right)	25	512*512	40	5	10576
S16	25	512*512	10	5	2644

S17	25	256*256	10	3	678
S18B (top)	25	256*256	40	3	2712
S18B (middle)	25	256*256	100	4	6780
S18B (bottom)	25	256*256	250	5	16950
S18D	25	256*256	10	3	678
S19 (related to 3A-B)	25	256*256	785	3	53223
S20A	25	256*256	204	3	13831
S20C	25	256*256	631	3	42782
S21 (related to 3F)	3	1024*1024	1	5	1048
S22	5	256*256	20	5	1356
S23E	5	256*256	20	2	1356
S24A-B	10	1024*1024	21	5	22043
S24C	10	1024*1024	20	5	20960
S32	15	256*256	40	2	2712
S35C (left)	25	256*256	400	3	27120
S35C (right)	5	256*256	10	5	678
Movies S1 and S2 (related to 3A-B and S19)	25	256*256	785	3	53223
Movie S3 (related to 3D)	25	256*256	400	3	27120
Movie S4 (related to 1A)	25	512*512	160	5	41966

Supplementary Note 1. Limit of detection estimation for MoC-Abs.

An antibody has about 4 to 6 oligonucleotide binding sites if using maleimide-thiol conjugation. Each oligonucleotide used for standard MoC-Abs contains 12 isotopically-labeled nucleotides for a total of 48 to 72 isotopic labels per antibody (or an average of 60 atoms). Utilizing a cesium primary ion beam under our imaging conditions of 3 pA current and 1 ms dwell time per pixel, an approximate of 18,750 cesium ions arrive within a voxel of size 50,000 nm^3 (100 nm X, 100 nm Y and 5 nm Z). Assuming a conservative average estimate of 10 MoC-Abs atoms liberated per cesium ion (*3*), up to 187,500 MoC-Abs atoms per voxel is the upper bound of MoC-Ab atoms liberated. With an useful yield of 10% and an ion extraction efficiency of 95%, and following previously established assumptions of 3,000,000 atoms present within each voxel (*3*), an average of 0.375 ions are detected per antibody on each voxel. In other words, 2.7 MoC-Abs in a voxel would result in the detection of 1 elemental ion count on that voxel. Increasing the number of isotopically-labeled nucleotides per oligonucleotide from 12 to 40 ("Long MoC-Ab") would result in the detection of 1 elemental ion count per antibody.

Current	3	**pA**
Dwell Time per voxel	1	**ms**
Cs+ Ions per Amp per s	6.25E+18	**Cs+**
Cs+ Ions impacting each analyzed voxel	18750	**Cs+ per each voxel**
Voxel size (X * Y * Z)	100 * 100 * 5	**nm^3**
Average sputtered MoC-Ab atoms per cesium ion	10	**atoms per Cs+**
Total Possible Sputtered MoC-Ab atoms per voxel	187500	**atoms per voxel**
Average labeled element atoms per MoC-Ab (standard)	60	**atoms**
Average labeled element atoms per MoC-Ab (long)	200	**atoms**
Useful Yield (ionized atom per sputtered atom)	0.1	**ions**
Ion Extraction Effciency	0.95	
Atomic Density/um^3	60000000000	**atoms per um^3**
Atomic Density/voxel	3000000	**atoms per voxel**
**For the Standard MoC-Ab**		
Average number of labeled element ions detected in 1 MoC-Ab in 1 voxel	0.375	**ions**
Average number of MoC-Abs to get 1 element ion count	2.7	**MoC-Abs**
**For the Long MoC-Ab**		
Average number of labeled element ions detected in 1 MoC-Ab in 1 voxel	1.25	**ions**
Average number of MoC-Abs to get 1 element ion count	0.8	**MoC-Abs**

Supplementary Note 2. Isotope barcoding

Assuming single antibody detection, combinatorial approaches might provide to the 7 detectors design an increase on the absolute number of detected biomolecules. For example, the isotopes ^{19}F, ^{76}Se, ^{79}Br, ^{81}Br, ^{122}Te, ^{125}Te and ^{127}I could be leveraged for the synthesis of nucleoside phosphoramidites. These reagents would be combined in groups of three to generate 35 unique mass-oligonucleotides (**Figure Note 2**). Each mass-oligonucleotide could be conjugated to a distinct antibody. Despite other more complicated strategies could be leveraged for barcoding, an n-choose-k would ensure that only regions in 3D space with three of the seven isotopes will be selected as real signal.

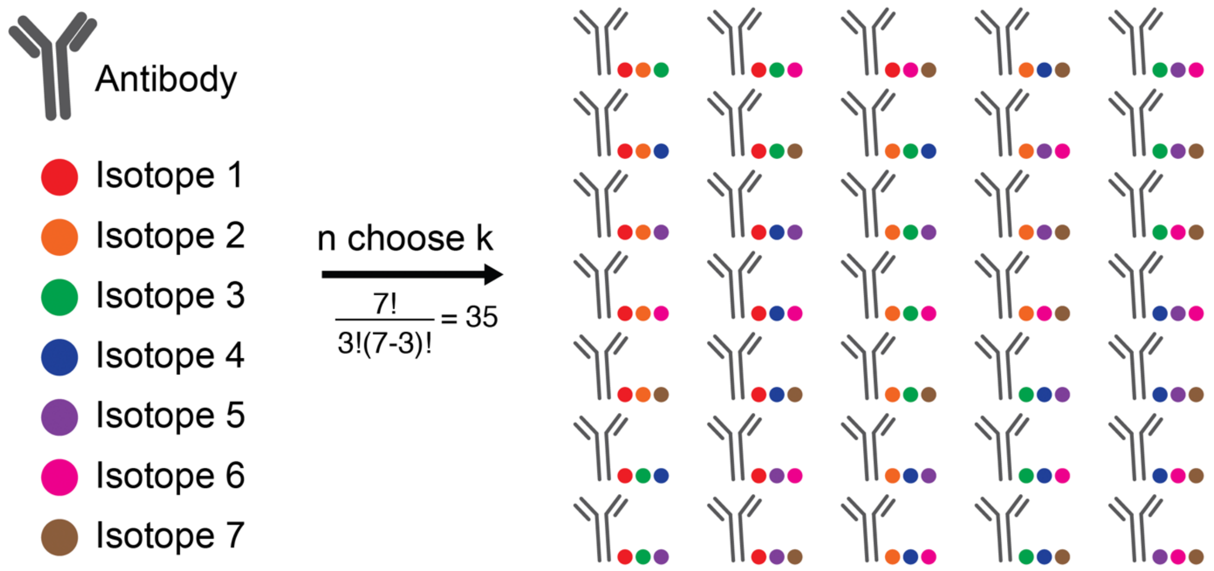

Figure Note 2. Schematic representation of isotope barcoding. Antibodies would be conjugated to mass-oligonucleotides synthesized with three of the seven isotope-derivatized nucleotides for a total of 35 combinations.

Supplementary Note 3. HD-MIBI: complexity, expenses and availability

Ion beam imaging is a method that uses isotopically labeled antibodies to target proteins in biological samples and detect them by secondary ion mass spectrometry. In the Nolan lab, we routinely study tissue architecture using the MIBIscope (Ionpath, Inc), which allows to detect >40 proteins in tissue sections using antibodies conjugated to lanthanides via a polymer with several DTPA chelators. While the configurations of the MIBIscope are excellent for the fast and simultaneous acquisition of large fields of view in tissue sections, it is currently hindered by its lateral and axial resolution. The NanoSIMS (CAMECA, Inc) can be equipped with a cesium gun with a finer spot size; thus offering an improved spatial resolution (despite its more limited field of view). The lanthanide isotopes used in the MIBIscope are not easily ionized by the cesium gun, thus inspiring this work in developing MoC-Abs enable protein imaging in biological samples, using secondary ion mass spectrometry, at resolutions akin to what can be obtained using super-resolution optical microscopy techniques.

Running a HD-MIBI experiment requires 1) MoC-Abs assembly, 2) cell staining and 3) data acquisition by NanoSIMS:

1. MoC-Abs assembly

Oligonucleotide synthesis often requires specialized equipment; therefore, the optimal option is to order them from companies specializing in this. As an example, the synthesis cost for the bromine oligonucleotide (described in Supplementary Table 1) is around 1,200.00 USD for the 200 nmol scale (Genelink, Inc), which is enough for >10 conjugations to 50 μg of antibody each. Oligonucleotide conjugation to antibodies is based on maleimide reaction chemistry. Therefore, antibodies need to be free of protein carriers such as BSA. Custom orders to obtain purified, carrier

free antibodies may increase the cost of these reagents. Materials and equipment for maleimide deprotection, lyophilization and conjugation to antibodies are commonly found in standard molecular biology laboratories worldwide.

2. Cell staining

Materials and equipment for HD-MIBI staining are commonly found in standard molecular biology laboratories worldwide.

3. Data acquisition by NanoSIMS

The NanoSIMS is a dedicated instrument, usually found in core facilities at major research institutions. Data acquisition is complex and often requires a trained engineer to operate the instrument. Expenses may vary between institutions, for example, the 2019 fees at Stanford University to run the NanoSIMS are 90/45 USD (Peak/Off-peak hourly rate) for academia and non-profit organizations, and 270/135 USD (Peak/Off-peak hourly rate) for industry and other organizations. There are >45 NanoSIMS available in major academic or governmental institutions around the world, with multiple locations in Asia, Europe and North America (**Map 1**).

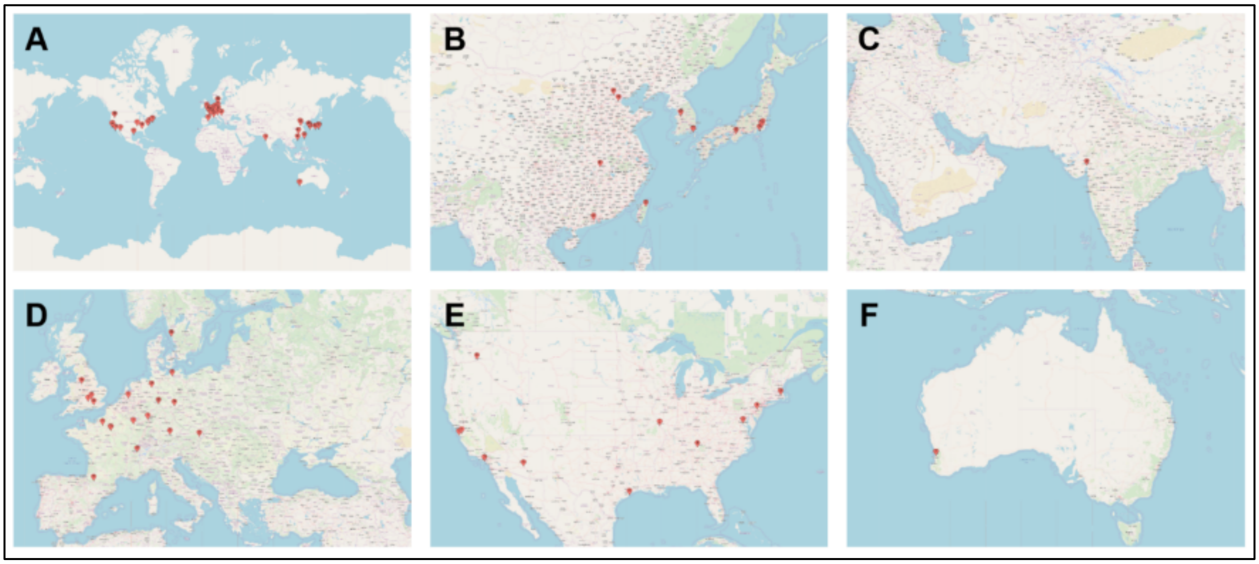

Map 1. NanoSIMS locations. (**A**) Worldwide, (**B-C**) Asia, (**D**) Europe, (**E**) North America and (**F**) Australia. Maps Data: Google, ©2019

A non-exhaustive list of experts in the use of nanoSIMS for biological applications:

Prof. Garry P Nolan, Dr. Mike Angelo and Mr. Chuck Hitzman (Stanford, USA), Prof. Claude Lechene and Dr. Matthew Steinhauser (Harvard Medical School, USA), Dr. Peter K Weber and Dr. Jennifer Pett-Ridge (Lawrence Livermore National Laboratory, USA), Prof. Peter Williams (Arizona State University), Dr. Zihua Zhu (Pacific Northwest National Laboratory, USA), Dr. Jean-Luc Guerquin-Kern (Institut Curie Recherche, France), Prof. Chris RM Grovenor (University of Oxford, UK), Prof. Philippe Pareige (GPM-Rouen University, France), Dr. Maren Voss and Dr. Angela Vogst (IOW – Leibniz Institut fur Ostseeforschung Warnemunde, Germany), Prof, Jack Middelburg (Utrecht University, The Netherlands), Prof. Anders Meibom (EPFL, Switzerland), Dr. Hans-Hermann Richnow (Helmholtz Center for Environmental Research UFZ, Germany), Dr. Dirk Schaumloffel (IPREM Pau, France), Prof. Andrew Ewing and Dr. Per Malmberg (University of Gothenburg, Sweden), Prof. Silvio O Rizzoli (University Medical Center Gottingen, Germany), Prof. Ian Gilmore (National Physical Laboratory, UK), Dr. Greg McMahon (National Physical Laboratory, UK), Prof. Tom Wirtz (Luxembourg Institute of Science and Technology, Luxembourg), Dr. Marcel Kuypers and Dr. Sten Littmann (Max Planck Institute for Marine biology, Germany), Prof. Michael Wagner and Prof. Andreas Richter (University of Wien, Austria), Dr. Yuji Sano and Dr. Akira Isogai (University of Tokyo, Japan), Dr. Yuki Morono (JAMSTEC, Japan), Prof. Xiao-ping Xia (GIGCAS, China), Prof. Zhong-qiang Chen (China University of Geosciences, China), Prof. Yunchao Lang (Institute of Surface-Earth System Science, China) and Prof. Matt Kilburn (University of Western Australia, Australia).

Supplementary References

1. J. Jiang, J. Sheng, N. Carrasco, Z. Huang, Selenium derivatization of nucleic acids for crystallography. *Nucleic Acids Research* **35**, 477-485 (2007).
2. J. Sheng *et al.*, Synthesis, structure and imaging of oligodeoxyribonucleotides with tellurium-nucleobase derivatization. *Nucleic Acids Research* **39**, 3962-3971 (2011).
3. C. Lechene *et al.*, High-resolution quantitative imaging of mammalian and bacterial cells using stable isotope mass spectrometry. *Journal of Biology* **5**, 20 (2006).
