# Supplementary material for: Subcellular localization of biomolecules and drug distribution by high-definition ion beam imaging
Source: Nat Commun. 2021 Jul 30;12:4628. doi: 10.1038/s41467-021-24822-1 (PMC8324837; doi:10.1038/s41467-021-24822-1)
Supplement: Supplementary file 2 — Description of Additional Supplementary Files [file 41467_2021_24822_MOESM2_ESM.pdf]

Title: Supplementary Movie 1. 3D reconstruction of nucleoli from a single cell.

Description: 3D surface reconstruction of the nucleoli from a single HeLa cell. HeLa cells were stained with anti-nucleolin-^{19}F/FITC, and 785 individual planes were acquired to obtain srIBI images of a nucleolus from its appearance to its disappearance.

Title: Supplementary Movie 2. 3D reconstruction of a single nucleolus.

Description: 3D surface reconstruction of a nucleolus shown in Fig. 3B and Movie S1. HeLa cells were stained with anti-nucleolin-^{19}F/FITC, and 785 individual planes were acquired to obtain srIBI images of a nucleolus from its appearance to its disappearance. See Fig. S17 for images of each individual plane.

Title: Supplementary Movie 3. 3D reconstruction of a single cell.

Description: Representative 3D reconstruction of nucleolin (cyan), phosphorus (blue), H3K9me3 (magenta), H3K27Ac (green), and SC35 (red) in a HeLa cell stained with anti-nucleolin-^{19}F/FITC, anti-H3K9me3-^{81}Br/Cy3, anti-H3K27Ac-^{127}I/Cy5, and anti-SC35-biotin (recognized by streptavidin-^{197}Au/FITC). The image consists of the 3D reconstruction of a stack of 400 consecutive planes.

Title: Supplementary Movie 4. 3D reconstruction of a single cell treated with cisplatin.

Description: Representative 3D reconstruction of carbon (grey), nucleolin (cyan), phosphorus (blue), H3K9me3 (magenta), H3K27Ac (green), cisplatin (yellow), and SC35 (red) in a TYK-nu cell treated with 5 µM cisplatin for 24 hours and stained with anti-nucleolin-^{19}F/FITC, anti-H3K9me3-^{81}Br/Cy3, anti-H3K27Ac-^{127}I/Cy5, and anti-SC35-biotin (detected with streptavidin-^{197}Au/FITC). The image consists of the 3D reconstruction of a stack of 160 consecutive planes.
