# Supplementary material for: Subcellular localization of biomolecules and drug distribution by high-definition ion beam imaging
Source: Nat Commun. 2021 Jul 30;12:4628. doi: 10.1038/s41467-021-24822-1 (PMC8324837; doi:10.1038/s41467-021-24822-1)
Supplement: Supplementary file 3 — Reporting Summary [file 41467_2021_24822_MOESM3_ESM.pdf]

nature research

Corresponding author(s): Garry P Nolan

Last updated by author(s): May 10, 2021

Reporting Summary

Nature Research wishes to improve the reproducibility of the work that we publish. This form provides structure for consistency and transparency in reporting. For further information on Nature Research policies, see our Editorial Policies and the Editorial Policy Checklist.

Statistics

For all statistical analyses, confirm that the following items are present in the figure legend, table legend, main text, or Methods section.

n/a	Confirmed	
☐	☒	The exact sample size (*n*) for each experimental group/condition, given as a discrete number and unit of measurement
☐	☒	A statement on whether measurements were taken from distinct samples or whether the same sample was measured repeatedly
☐	☒	The statistical test(s) used AND whether they are one- or two-sided<br>*Only common tests should be described solely by name; describe more complex techniques in the Methods section.*
☒	☐	A description of all covariates tested
☐	☒	A description of any assumptions or corrections, such as tests of normality and adjustment for multiple comparisons
☐	☒	A full description of the statistical parameters including central tendency (e.g. means) or other basic estimates (e.g. regression coefficient) AND variation (e.g. standard deviation) or associated estimates of uncertainty (e.g. confidence intervals)
☐	☒	For null hypothesis testing, the test statistic (e.g. *F*, *t*, *r*) with confidence intervals, effect sizes, degrees of freedom and *P* value noted<br>*Give P values as exact values whenever suitable.*
☒	☐	For Bayesian analysis, information on the choice of priors and Markov chain Monte Carlo settings
☒	☐	For hierarchical and complex designs, identification of the appropriate level for tests and full reporting of outcomes
☒	☐	Estimates of effect sizes (e.g. Cohen's *d*, Pearson's *r*), indicating how they were calculated

Our web collection on statistics for biologists contains articles on many of the points above.

Software and code

Policy information about availability of computer code

Data collection	For ion beam imaging experiments, the software used to collect the data was NanoSIMS 50 from Cameca.<br>For fluorescence microscopy experiments, the software used to collect the data was Zen Black v2.1 from Zeiss and the analysis software for BZ-X microscopes from Keyence.<br>For mass cytometry experiments, the software used to collect the data was DVS Sciences Cytof instrument control software from Fluidigm.
Data analysis	We used the ImageJ plugin openMIMS (github.com/BWHCNI/OpenMIMS) to open nanoSIMS images. We used FIJI (version 2.0.0-rc-68/1.52h) to sum several planes and apply gaussian blur. We used Imaris v9.2 from Oxford Instruments for three dimensional rendering of ion beam imaging data. We used open source R 3.6.2) for data analysis. FASTQC v.0.11.8, Trim Galore v.0.6.0, and HISATv2.1.0 for RNA-seq data processing. We used Cytobank for data visualization of mass cytometry experiments. We used Adobe Illustrator (version 24.0.3) for viewing images and assembling images, graphs, drawings and text into figure panels.

For manuscripts utilizing custom algorithms or software that are central to the research but not yet described in published literature, software must be made available to editors and reviewers. We strongly encourage code deposition in a community repository (e.g. GitHub). See the Nature Research guidelines for submitting code & software for further information.

Data

Policy information about availability of data

All manuscripts must include a data availability statement. This statement should provide the following information, where applicable:

- Accession codes, unique identifiers, or web links for publicly available datasets
- A list of figures that have associated raw data
- A description of any restrictions on data availability

All data is available in the main text or the supplementary materials. Relevant data generated and analyzed during the current study are available from the

corresponding author on reasonable request. All sequencing data is deposited to GEO under Accession Number GSE141138 [https://www.ncbi.nlm.nih.gov/geo/query/acc.cgi?acc=GSE141138]. The human genome (GRCh38.98) is from Ensembl [http://ftp.ensembl.org/pub/release-98/gtf/homo_sapiens/]. The alignment index for HISAT2 is [https://genome-idx.s3.amazonaws.com/hisat/grch38_genome.tar.gz].

Field-specific reporting

Please select the one below that is the best fit for your research. If you are not sure, read the appropriate sections before making your selection.

[x] Life sciences [ ] Behavioural & social sciences [ ] Ecological, evolutionary & environmental sciences

For a reference copy of the document with all sections, see nature.com/documents/nr-reporting-summary-flat.pdf

Life sciences study design

All studies must disclose on these points even when the disclosure is negative.

Sample size	A substantial amount of the experiments performed in this manuscript are aimed at describing a new technology, therefore, no sample size calculation was performed. Sample size was selected until we properly demonstrate and validate technical performance. For biological experiments, the sample size (n) were reported as an exact number. No statistical methods were used to predetermine sample size.
Data exclusions	No data were excluded.
Replication	All attempts of replication were successful. All experiments have been repeated independently three or more times with similar results, unless specified in the figure legend.
Randomization	Randomization was not necessary for this study. Randomization is not relevant for this study because the experiments were aiming to validate technical performance.
Blinding	The investigators were not blinded to group allocation during data collection and analysis. Blinding is not relevant for this study because the experiments were aiming to validate technical performance. For confocal microscopy and ion beam imaging experiments, fields of view for each group were randomly selected and images recorded. Representative images were shown.

Reporting for specific materials, systems and methods

We require information from authors about some types of materials, experimental systems and methods used in many studies. Here, indicate whether each material, system or method listed is relevant to your study. If you are not sure if a list item applies to your research, read the appropriate section before selecting a response.

Materials & experimental systems

n/a	Involved in the study	
[ ]	[x]	Antibodies
[ ]	[x]	Eukaryotic cell lines
[x]	[ ]	Palaeontology and archaeology
[x]	[ ]	Animals and other organisms
[ ]	[x]	Human research participants
[x]	[ ]	Clinical data
[x]	[ ]	Dual use research of concern

Methods

n/a	Involved in the study	
[x]	[ ]	ChIP-seq
[x]	[ ]	Flow cytometry
[x]	[ ]	MRI-based neuroimaging

Antibodies

Antibodies used

Information of the antibodies used in this study is also detailed in supplementary table.
The isotope-conjugated primary antibodies were:
dsDNA --> catalog number ab27156 from Abcam clone 35I9, tag 162Dy, staining concentration 1:50
dsDNA --> catalog number ab27156 from Abcam clone 35I9, tag 127I, staining concentration 1:50
nucleolin --> catalog number ADI-KAM-CP100-E from Enzo clone 4E2, tag 19F / 6-FAM, staining concentration 1:100
H3K27Ac --> catalog number ab4729 from Abcam clone pAb, tag 19F / 6-FAM, staining concentration 1:100
CENP-A --> catalog number ab13939 from Abcam clone 3-19, tag 81Br / Cy3, staining concentration 1:500
H3K27Ac --> catalog number ab4729 from Abcam clone pAb, tag 127I / Cy5, staining concentration 1:100
SC35 --> catalog number 556363 from BD Biosciences clone αSC35, tag Biotin, staining concentration 1:100
H3K9me3 --> catalog number ab8898 from Abcam clone pAb, tag 81Br / Cy3, staining concentration 1:100
nucleolin --> catalog number ADI-KAM-CP100-E from Enzo clone 4E2, tag 127I / Cy5, staining concentration 1:100
NPM1 --> catalog number MABE937 from EMD Millipore clone 28M1, tag Biotin, staining concentration 1:100
FBL --> catalog number MABE1154 from EMD Millipore clone 38F3, tag 81Br / Cy3, staining concentration 1:50

The fluorophore-conjugated secondary antibodies were:

	anti-mouse IgG-alexa488 --> catalog number 4408S from CST<br>anti-mouse IgG-alexa647 --> catalog number 4410S from CST<br>anti-rabbit IgG-alexa647 --> catalog number 4414S from CST
Validation	All antibodies used in this study were individually validated and titrated. The expected patterns for all of them were obtained before and after MoC-Ab conjugation. All targets have distinctive patterns and we used multiplexed imaging as counterstain.

Eukaryotic cell lines

Policy information about cell lines

Cell line source(s)	HeLa (ATCC), Jurkat (Clone E6.1; ATCC) and TYK-nu cells (a gift from Dr. Fantl at Stanford).
Authentication	None of the cell lines used were authenticated.
Mycoplasma contamination	All cell lines were tested negative for Mycoplasma contamination.
Commonly misidentified lines<br>(See ICLAC register)	No commonly misidentified cell lines were used in this study.

Human research participants

Policy information about studies involving human research participants

Population characteristics	We have only used one frozen skin OCT block in this study to demonstrate srIBI application to tissue sections. No biological conclusions were obtained from these experiments and therefore the population characteristics are not relevant.
Recruitment	Not applicable to our study as described in the section "population characteristics".
Ethics oversight	The frozen skin OCT block used in this study was collected under a protocol approved by the Institutional Review Board at Stanford University (protocol no. 35324). Individuals donating fresh surgical tissue provided informed consent.

Note that full information on the approval of the study protocol must also be provided in the manuscript.
